# Transient receptor potential vanilloid 2 functions as a directional driver for hepoxilin A_3_–mediated neutrophil migration

**DOI:** 10.1126/sciadv.adz1986

**Published:** 2026-07-31

**Authors:** Claudia Feriotti, Regino Mercado-Lubo, Merran Bryford, Rebecca Ferrisi, Francesca Gado, Clementina Manera, Keyi Liu, Ping Lu, Yushuan Lai, Beth A. McCormick, Randall Mrsny

**Affiliations:** ^1^ Department of Life Sciences, Center for Therapeutic Innovation, University of Bath, Claverton Down, Bath BA2 7AY, UK.; ^2^ Department of Microbiology, University of Massachusetts Chan Medical School, Worcester, MA 01605, USA.; ^3^ Dipartimento di Farmacia, Università di Pisa, Via Bonanno 6, 56126 Pisa, Italy.

## Abstract

Neutrophil migration involves a dynamic balance of chemoattractant and inhibitory signals. We previously identified hepoxilin A_3_ (HxA_3_) as a potent chemoattractant that directs neutrophil migration across intestinal epithelia and showed that endocannabinoids can suppress migration through the cannabinoid 2 receptor (CB_2_R). Here, we reveal that HxA_3_ uses the transient receptor potential vanilloid type 2 (TRPV2) cation channel to promote transmigration, a process finely tuned by physical TRPV2-CB_2_R interaction. In resting neutrophils, surface-localized CB_2_R responding to endocannabinoids suppresses migration, while a small fraction of TRPV2 at the plasma membrane “senses” HxA_3_. HxA_3_ drives the movement of an intracellular “storage” pool of TRPV2 to the leading edge of migration where physical interaction with CB_2_R establishes cell surface receptor complexes to direct neutrophil migration. Postactivation, TRPV2 is shunted toward degradation, while CB_2_R is both replaced and recycled to the plasma membrane to establish a coordinated system capable of maintaining directional fidelity to HxA_3_ gradients. These findings uncover a previously unrecognized layer of neutrophil trafficking regulation.

## Teaser

Neutrophils use a dual receptor complex to navigate to sites of intestinal inflammation.

## INTRODUCTION

The luminal environment of the intestine normally coexists in harmony with a complex array of microbes, but it must also be poised to rapidly respond to enteric pathogens (*1*). Neutrophils play a pivotal role as first line defenders against luminal insults sensed by the intestinal mucosa, being called from the vasculature into the lamina propria through a process known as diapedesis in response to interleukin-8 (IL-8) being released from the basolateral surface of intestinal epithelial cells (*2*). To reach the intestinal lumen, neutrophils must undergo a second migratory step, navigating from the lamina propria through the paracellular spaces between adjacent epithelial cells (*3*). Neutrophils then fully activate in the lumen to release a plethora of lytic and toxic materials to combat pathogens (*4*). There is a conundrum, however, of how neutrophils can navigate the lamina propria to reach the intestinal lumen through directed migration without functional transformation to a phenotype capable of releasing lytic and toxic materials to combat pathogens, events that would cause intestinal tissue damage and limit neutrophil access to luminal pathogenic targets.

We previously demonstrated hepoxilin A_3_ (HxA_3_) as a pure chemoattractant used by neutrophils to cross from the lamina propria to access the intestinal lumen by migrating between adjacent epithelial cells at sites of inflammation (*5*). Further, we showed that inflammatory triggers induce the up-regulation of an apically expressed adenosine triphosphate–binding cassette (ABC) family transporter, multidrug resistance protein 2 (MRP2), that secretes HxA_3_ into the intestinal lumen to establish the required gradient of this chemoattractant to draw neutrophils to this site (*6*). As a critical counterbalance to the HxA_3_/MRP2 axis, we demonstrated a role for another ABC transporter, P-glycoprotein (P-gp) positioned at the apical surface of intestinal epithelial cells. Previously appreciated for its capacity to efflux xenobiotics, we identified endocannabinoids (eCBs) as the first endogenous substrate for P-gp and showed how eCBs could suppress the chemoattractant actions of HxA_3_ on neutrophils (*7*). Understanding how these opposing signaling axes of HxA_3_/MRP2 and P-gp/eCBs could control neutrophil migration have shed light on both acute inflammatory processes, such as gut inflammation induced by enteric pathogens, and chronic inflammatory conditions, where intestinal biopsies from patients with active inflammatory bowel disease (IBD) show reduced P-gp/MRP2 expression ratios (*6*, *8*).

Despite these discoveries, much remains unknown about the opposing mechanisms of action by which eCBs and HxA_3_ counterbalance neutrophil migratory signaling. Neutrophils express two cannabinoid receptors, CB_1_R and CB_2_R (*9*), with the latter shown to suppress HxA_3_-mediated transepithelial migration (*7*). HxA_3_ can directly bind to neutrophil membranes (*10*) and mobilize Ca^2+^ within neutrophils (*11*), suggesting a potential mechanism for this eicosanoid lipid to mediate directional neutrophil migration. To establish and maintain directional fidelity, however, a cell surface receptor that is rapidly internalized with continual presentation of nascent receptors to constantly sense changes in chemotactic gradient would be required, as has been shown for other migrating cells (*12*, *13*). We used this concept to develop a proteomic screen of rapidly internalized cell surface proteins induced by HxA_3_ to identify the transient receptor potential (TRP) cation channel subfamily V member 2 or vanilloid type 2 (TRPV2) as the HxA_3_-activated neutrophil cell surface receptor involved in the directional migration of these cells. We also show that the cannabinoid receptor CB_2_R associates with TRPV2 and regulates its expression and function at the plasma membrane to sense HxA_3_. Together, our findings reveal interactive and coordinated activities between CB_2_R and TRPV2 that are needed for HxA_3_-mediated neutrophil migration, identifying a previously unrecognized paradigm of neutrophil migration, in which inhibitory and stimulatory components coalesce into a cell surface complex that enables rapid responses to changing chemotactic gradients.

## RESULTS

### TRPV2 identified as neutrophil receptor mediating HxA_3_-induced chemotaxis

**Fig. 1.**
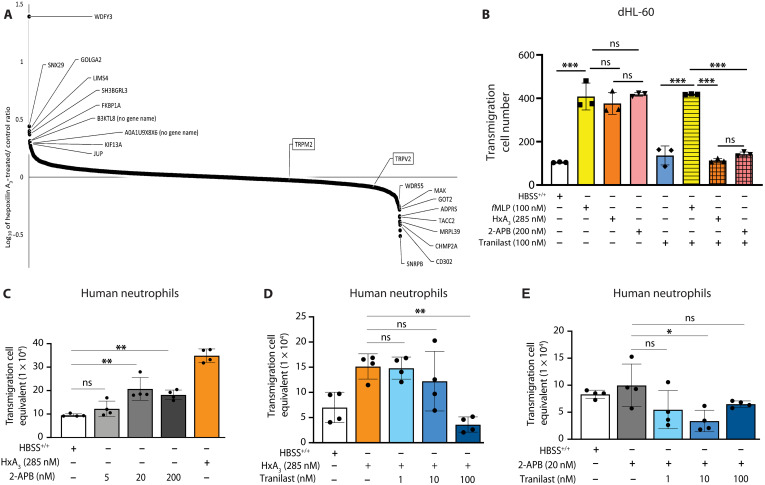
TRPV2 was identified as the potential cell surface receptor for HxA_3_. (**A**) Intensity plot of proteins identified by proteomic analysis of sample collected using the cell surface subtraction schema (fig. S2). (**B**) The nonselective TRPV agonist 2-aminoethoxydiphenyl borate (2-APB) at 200 nM was tested for induction of dHL-60 cell migration compared to HxA_3_ and *f*MLP. TRPV2 channel blocker *N*-(3′,4′-dimethoxycinnamoyl) anthranilic acid (tranilast) at 100 nM was then tested for suppression of dHL-60 cell migration induced by HxA_3,_ 2-APB, or *f*MLP. Migration studies for HL-60 and dHL-60 cells were performed for 30 min at 37°C using Transwell filters. Data are shown as means ± SEM for at least three independent experiments (*n* = 3) for dHL-60 cells [(A) and (B)]. (**C** to **E**) Migration phenotypes were confirmed using freshly isolated human neutrophils in the exact same experimental procedures. Shown are representative data from single donors of at least three independent experiments (*n* > 3). Statistical analysis performed by one-way ANOVA with Bonferroni correction for multiple comparisons. ***P < 0.001; **P < 0.01; *P < 0.05; not significant (ns), *P* > 0.05.

To identify the neutrophil receptor for HxA_3_, we developed a capture and enrichment assay using the human promyeloblast cell line HL-60 differentiated (dHL-60) into a neutrophil-like phenotype (*14*, *15*). We verified dHL-60 differentiation using the neutrophil cell surface marker CD11b (fig. S1A) and functional assays. While both HL-60 and dHL-60 cells migrated in response to the bacteria-derived *N*-formyl-Met-Leu-Phe (*f*MLP), HxA_3_-mediated migration was observed only with dHL-60 cells (fig. S1B). The chemically labile nature of HxA_3_ confounded efforts using traditional bait-capture methods to identify potential cell surface receptor(s) activated by HxA_3_. As directional chemotaxis should require cycles of cell surface receptor removal and replacement to provide a mechanism for continual sensing of a chemotactic agent along a concentration gradient to ensure directional fidelity, a cell surface enrichment capture analysis assay was performed where dHL-60 cells were surface biotinylated and then exposed to freshly prepared HxA_3_ to induce activation/internalization of any potential receptor(s). After 15 min, biotinylated proteins remaining at the cell surface were conjugated with streptavidin and removed following cell lysis. Internalized biotinylated receptors were then captured by a second streptavidin-bead conjugation step (fig. S2). Cell surface protein(s) that were increased or decreased in response to HxA_3_ treatment were identified by proteomics (Fig. 1A).

Notably, no established *f*MLP receptor was identified in this screen, consistent with the distinct functions of HxA_3_ and *f*MLP. Since HxA_3_ has previously been shown to induce cellular Ca^2+^ signaling, our attention focused on TRP channels, a family of highly conserved cation channels, with two being identified in our screen: TRP melastatin 2 (TRPM2) and TRPV2 (Fig. 1A). TRPM2 activation in neutrophils results in Ca^2+^ influx currents and appears involved in neutrophil activation functions such as NETosis, reactive oxygen species production, and bactericidal activities (*16*, *17*), making TRPM2 less likely to be the receptor activated by HxA_3_, which acts as a pure chemoattractant. Furthermore, *trpm2*^−/−^ mice showed increased neutrophil migration in response to *Listeria* infection but increased disease susceptibility (*18*). A role for TRPV2 was validated using the channel blocker *N*-(3′,4′-dimethoxycinnamoyl) anthranilic acid (tranilast) that can suppress neutrophil migration induced by lipopolysaccharide (*19*). Treatment with tranilast blocked HxA_3_ but not *f*MLP-induced neutrophil migration (Fig. 1B). Because of the labile nature of HxA_3_, we also tested the nonspecific channel agonist 2-aminoethoxydiphenyl borate (2-APB) to provide a surrogate to HxA_3_ in certain studies. Although treatment with *f*MLP, HxA_3_, or 2-APB induced dHL-60 migration, only HxA_3_ or 2-APB actions were blocked by tranilast (Fig. 1B). Dose-response studies were used to select concentrations of 2-APB and tranilast for in vitro migration studies (fig. S3, A and B) that were validated using freshly isolated human peripheral blood neutrophils (Fig. 1, C to E).

**Fig. 2.**
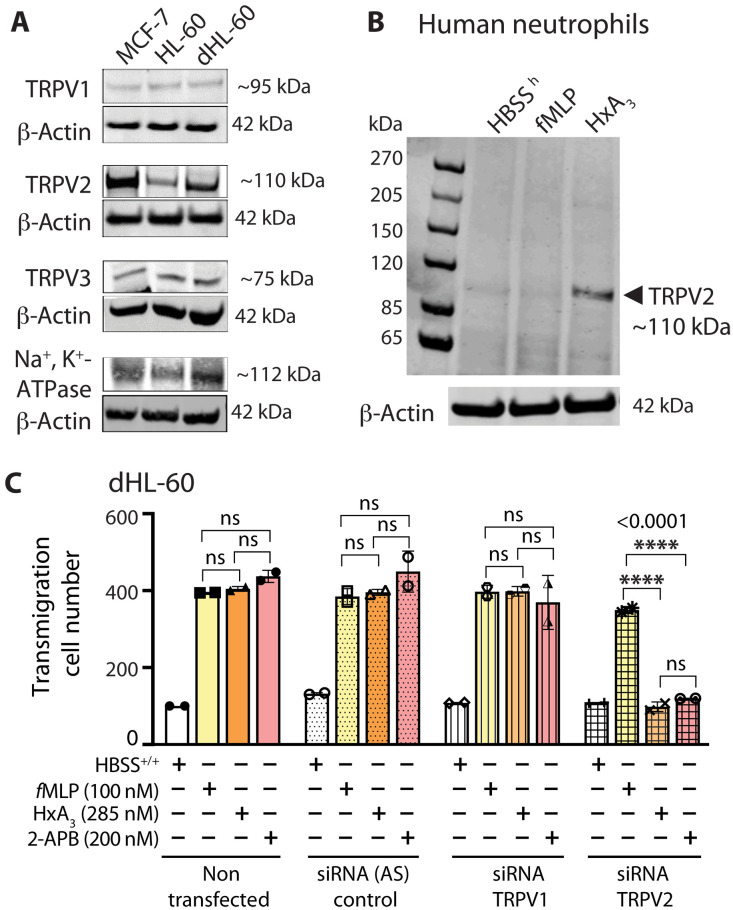
TRPV2 expression is up-regulated and required for migration of neutrophils in response to HxA_3_. (**A**) Western blot of 1 × 10^6^ nondifferentiated, dHL-60, or MCF-7 breast cancer cells showing relative expression levels of TRPV1, TRPV2, TRPV3, or Na^+^- and K^+^-dependent adenosine triphosphatase (Na^+^,K^+^-ATPase) (positive membrane protein control). (**B**) Freshly isolated human neutrophils probed for TRPV2 content after treatment with 100 nM *f*MLP or 285 nM HxA_3_ for 15 min. (**C**) Migration of dHL-60 cells induced by 100 nM *f*MLP, 285 nM HxA_3_, or 200 nM of the TRPV channel agonist 2-APB following 6 days of siRNA treatments to selectively knockdown TRPV1 or TRPV2 and corresponding AS controls. Migration studies for dHL-60 cells were performed for 30 min at 37°C using Transwell filters and compared to control buffer addition (HBSS^+/+^). Data are shown as means ± SEM for at least three independent experiments (*n* = 3). Statistical analysis performed by one-way ANOVA with Bonferroni correction for multiple comparisons. ****P < 0.0001; ns, *P* > 0.05. AS, antisense.

While 2-APB could replace HxA_3_ as a chemoattractant, it is also capable of nonselectively activating TRPV1, TRPV2, and TRPV3 (*20*). To confirm the specific involvement of TRPV2 in neutrophils, we compared expression of these channels. TRPV2 protein expression was increased in dHL-60 relative to HL-60, while TRPV1 and TRPV3 were unchanged (Fig. 2A). Moreover, human peripheral blood neutrophils, when stimulated with HxA_3_ but not with *f*MLP, induced TRPV2 protein expression (Fig. 2B), and both TRPV1 and TRPV3 were undetectable in primary human peripheral blood neutrophils (fig. S4A). We further confirmed the specificity of TRPV2 by small interfering RNA (siRNA)–mediated knockdown. Untreated dHL-60 cells and those treated with control siRNA or siRNA targeting TRPV1 all migrated in response to *f*MLP, HxA_3_, and 2-APB (Fig. 2C). dHL-60 cells treated with siRNA targeting TRPV2 retained responsiveness to *f*MLP but not HxA_3_ or 2-APB (Fig. 2C), confirming the specificity of TRPV2 and adding to the proteomic evidence that *f*MLP and HxA_3_ act on different receptors. Knockdowns by siRNAs were confirmed by quantitative polymerase chain reaction (qPCR; fig. S5).

### CB_2_R participates in TRPV2/HxA_3_-mediated neutrophil chemotaxis

**Fig. 3.**
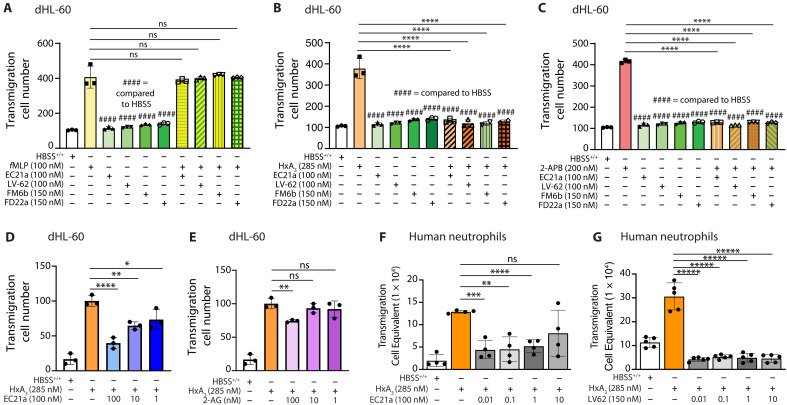
Activation of CB_2_R suppresses HxA_3_-induced neutrophil migration. Migration of dHL-60 cells induced by (**A**) 100 nM *f*MLP, (**B**) 285 nM HxA_3_, or (**C**) 200 nM of the TRPV channel agonist 2-APB compared to the control buffer addition (HBSS^+/+^) in the presence of the following CB_2_R agonists: 100 nM of positive allosteric modulator EC21a, 100 nM of orthosteric agonist LV-62, 150 nM of dual orthosteric agonist FM-6b, or 150 nM of dualsteric bitopic orthosteric/allosteric agonist FD22a. (**D**) EC21a and (**E**) 2-AG were tested for dose response to inhibition of HxA_3_-induced dHL-60 cell migration. Inhibition of HxA_3_-induced migration by EC21a (**F**) and LV-62 (**G**) appears not to be dose dependent in human neutrophils. Shown are representative results from a single donor [(F) and (G)] for at least three independent experiments. All migration studies (both dHL-60 cells and human neutrophils) were performed for 30 min at 37°C using Transwell filters. Data for dHL-60 cells are shown as means ± SEM for at least three independent experiments (*n* = 3). Statistical analysis performed by one-way ANOVA with Bonferroni correction for multiple comparisons. ****P < 0.0001; ***P < 0.001; **P < 0.01; *P < 0.05; ns, *P* > 0.05; ####*P* < 0.001 compared to negative control HBSS^+/+^.

HxA_3_-mediated neutrophil migration is opposed more by CB_2_R activation than CB_1_R activation, with the CB_1_R-specific eCB 2-arachidonoylglycerol (2-AG) being much less effective than *N*-acylethanolamine (NAE)–type eCB family members that can activate both CB_2_R and CB_1_R (*7*). To verify CB_2_R specificity in regulating HxA_3_-mediated migration, we tested a series of highly specific CB_2_R-activating agents: the positive allosteric modulator EC21a (100 nM), the orthosteric agonist LV-62 (100 nM), the dual orthosteric agonist FM-6b (150 nM), and the dualsteric bitopic orthosteric/allosteric agonist FD22a (150 nM) (*21*, *22*). All four of these specific CB_2_R-activating agents produced a small but statistically significant increase in dHL-60 cell migration compared to baseline migration activity in control Hanks’ balanced salt solution^+/+^ (HBSS^+/+^) treatment (Fig. 3, A to C), consistent with the potential for some cannabinoids to modulate TRPV2 structure and function (*23*). None of these CB_2_R-activating agents blocked dHL-60 migration induced by *f*MLP (Fig. 3A), but all blocked dHL-60 cell migration induced by HxA_3_ (Fig. 3B) and 2-APB (Fig. 3C). HxA_3_-mediated dHL-60 cell migration was inhibited by EC21a and 2-AG in a dose-dependent manner with EC21a (Fig. 3D) having a stronger effect than 2-AG (Fig. 3E), in line with the specificity of CB_2_R over CB_1_R in suppressing HxA_3_-induced neutrophil migration (*7*). Similar effects were observed with other NAE-type eCBs (fig. S6). EC21a and LV-62 also blocked HxA_3_-induced migration in freshly isolated human neutrophils (Fig. 3, F and G), although effective concentrations varied across donors and no dose dependency was observed.

**Fig. 4.**
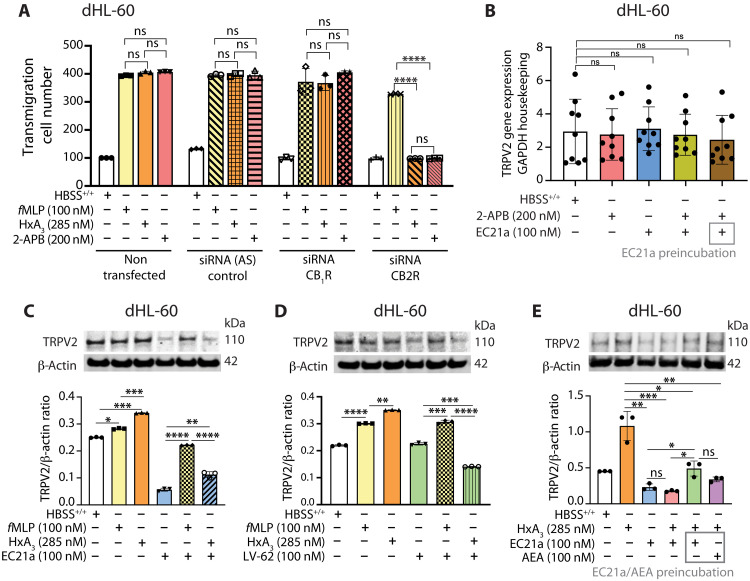
TRPV2 activity and expression is regulated by CB_2_R. (**A**) Migration of dHL-60 cells induced by 100 nM *f*MLP, 285 nM HxA_3_, or 200 nM of the TRPV channel agonist 2-APB compared to the control buffer addition (HBSS^+/+^) following 6 days of siRNA treatments targeting CB_1_R or CB_2_R. (**B**) qPCR of TRPV2 in dHL-60 cells after 15 min treatment with 2-APB, EC21a, both, or preincubation with EC21a, followed by 2-APB, compared to glyceraldehyde-3-phosphate dehydrogenase (GAPDH) as housekeeping gene. Total cellular protein expression of TRPV2 in dHL-60 cells (1 × 10^6^) after treatment with 100 nM *f*MLP or 285 nM HxA_3_, with or without 100 nM CB_2_R agonists (**C**) EC21a (positive CB_2_R allosteric modulator) or (**D**) LV-62 (the orthosteric CB_2_R agonist) compared to HBSS^+/+^. (**E**) Total cellular TRPV2 protein after 15-min treatment with 285 nM HxA_3_ with and without 100 nM EC21a compared to dHL-60 pretreated for 15 min with CB_2_R agonists EC21a or arachidonoyl ethanolamine (AEA), followed by 15-min treatment with HxA_3_ and HBSS^+/+^. Representative Western blots and averaged band densities as means ± SEM for at least three independent experiments (*n* = 3). Statistical analysis performed by one-way ANOVA with Bonferroni correction for multiple comparisons. ****P < 0.0001; ***P < 0.001; **P < 0.01; *P < 0.05; ns, *P* > 0.05.

Although activation of CB_2_R repressed HxA_3_-mediated dHL-60 migration, the presence of CB_2_R was paradoxically required for HxA_3_-mediated migration activity. When CB_2_R was knocked down by siRNA, we observed significant reduction of HxA_3_- and 2-APB–induced dHL-60 migration, comparable to HBSS^+/+^ control (Fig. 4A), while *f*MLP-induced migration remained. Treatment of dHL-60 cells with control siRNA or siRNA specific for CB_1_R showed no effect. These results implicate a more complex regulatory interplay between CB_2_R and TRPV2 activities, with CB_2_R possibly directly modulating TRPV2 activity while being able to also suppress HxA_3_ signaling.

We next examined the impact of CB_2_R activation on TRPV2 expression. We first verified that siRNA knockdown of TRPV2 was selective and did not alter TRPV1 gene expression (fig. S5A). While siRNA knockdown targeting TRPV1 markedly suppressed this gene, there was also a slight reduction in TRPV2 gene expression (fig. S5B). Activation of CB_2_R did not affect TRPV2 mRNA levels (Fig. 4B) but did alter total cellular protein levels of TRPV2 (Fig. 4, C to E). Treatment of dHL-60 cells with HxA_3_ resulted in greater TRPV2 total protein compared to treatment with *f*MLP (Fig. 4, C and D); treatment with both *f*MLP and HxA_3_ also increased TRPV2 total protein over HBSS^+/+^ control. Treatment with the CB_2_R-positive allosteric modulator EC21a caused substantial reduction of TRPV2 (Fig. 4C). Notably, reduction of TRPV2 levels caused by EC21a was less when *f*MLP was coadministered than with simultaneous treatment with HxA_3_. TRPV2 level changes induced by EC21a were replicated by LV-62, but its actions were not as marked (Fig. 4D). In comparison, total cellular TRPV1 levels in dHL-60 cells were unaffected by treatment with EC21a alone or when coadministered with HxA_3_ or *f*MLP (fig. S7). Preincubation with EC21a, simulating a baseline homeostatic environment, did not alter TRPV2 mRNA levels (Fig. 4B) but did lead to statistically higher TRPV2 protein levels compared to simultaneous coadministration of EC21a and HxA_3_ (Fig. 4E). These results not only are consistent with enhanced TRPV2 activity in HxA_3_-mediated cell migration but also suggest that CB_2_R activation further modulates TRPV2 expression and activity.

### TRPV2 redistributes to the neutrophil leading edge during HxA_3_-induced migration

**Fig. 5.**
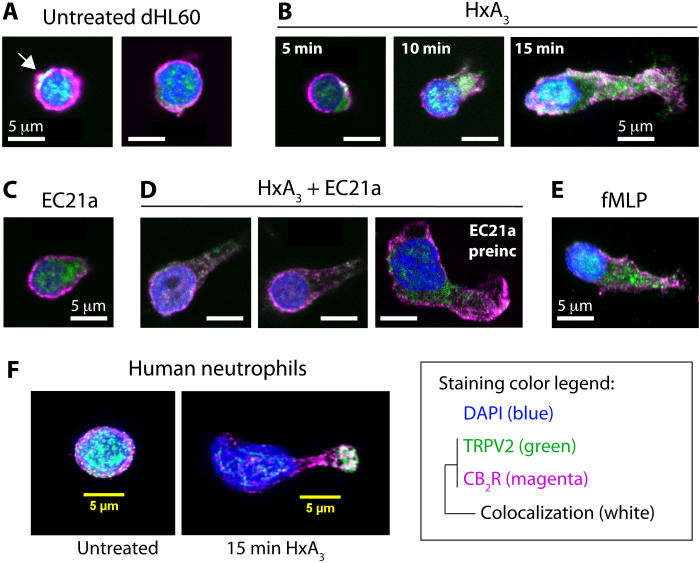
Cellular TRPV2 and CB_2_R distribution is altered in response to chemoattractant-induced polarization. Representative fluorescent microscopic images (single *z* planes) of dHL-60 cells demonstrating the subcellular location of TRPV2 (green) and CB_2_R (magenta) and their colocalization (white). Cells shown were treated for 15 min with (**A**) control buffer (HBSS^+/+^) or 285 nM HxA_3_ for (**B**) 5, 10, or 15 min. These cellular distributions of TRPV2 and CB_2_R were compared to dHL-60 cells treated for 15 min with a gradient of (**C**) 200 nM EC21a, (**D**) 200 nM EC21a plus 285 nM HxA_3_ simultaneously added or with 200 nM EC21a added 15 min before 285 nM HxA_3_ (right), and (**E**) 100 nM *f*MLP. Composite images use 4′,6-diamidino-2-phenylindole (DAPI) (blue) for nuclear staining. Scale bars, 5 μm.

Neutrophils undergo shape changes in response chemotactic factors, a process that aligns dynamic cytoskeletal events required for directional migration (*24*). This polarization, characterized by formation of a leading-edge pseudopod (*25*) and a trailing contractile element region defined as the uropod, can occur in the absence of a chemotactic gradient (*26*). At rest, CB_2_R was predominantly localized at the plasma membrane of dHL-60 cells; some TRPV2 was focally colocalized with CB_2_R at the plasma membrane (Fig. 5A, arrow), but most was present in intracellular vesicles adjacent to the nucleus. Treatment with HxA_3_ resulted in mobilization of intracellular TRPV2 (Fig. 5B). At 5 min post–HxA_3_ treatment, TRPV2 was observed in forming pseudopods and colocalized with CB_2_R at the leading-edge plasma membrane, events that were increased at 10 min. At 15 min, CB_2_R-TRPV2 colocalizations were present throughout the cell at or near the plasma membrane that were biased to the pseudopod region.

These observations in neutrophils are comparable to translocation of intracellular TRPV2 storage pools described in other cell types and in response to a variety of stimuli (*27*–*31*). What was not appreciated in these previous studies was potential regulation of this channel protein by a cannabinoid receptor. Treatment of dHL-60 cells with EC21a resulted in limited amounts of CB_2_R-TRPV2 colocalization at the plasma membrane but with occasional protrusions, similar to those observed in early pseudopods (Fig. 5C). Simultaneous treatment of dHL-60 cells with EC21a and HxA_3_ for 15 min resulted in a muted leading-edge pseudopod formation and minimal TRPV2-CB_2_R colocalizations (Fig. 5D). Pretreatment of dHL-60 cells with EC21a followed by HxA_3_ showed similar results. Treatment of dHL-60 cells for 15 min with *f*MLP induced pseudopods with TRPV2 occupying the pseudopod body but did not result in TRPV2-CB_2_R colocalizations (Fig. 5E). Studies in human neutrophils exposed to HxA_3_ affirm our findings in dHL-60 (Fig. 5F). These studies are consistent with TRPV2 and CB_2_R being positioned at the leading-edge pseudopod in response to HxA_3_ but not *f*MLP.

### HxA_3_ incites TRPV2 and CB_2_R to physically interact

**Fig. 6.**
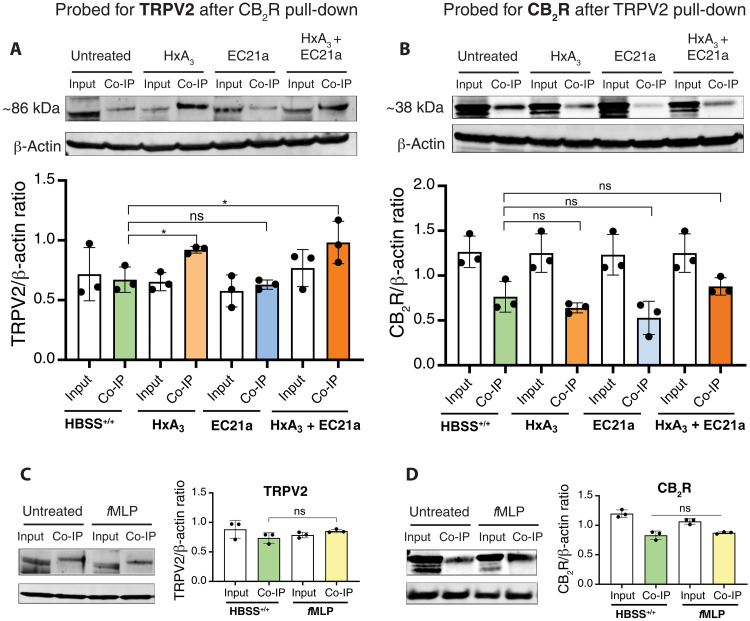
TRPV2 and CB_2_R physically interact, with these associations affected by HxA_3_ and CB_2_R activation. Representative Western blots of coimmunoprecipitation (Co-IP) analyses are shown where (**A**) a pull-down of CB_2_R was probed for the presence TRPV2 or (**B**) a pull-down of TRPV2 was probed for the presence of CB_2_R. Densitometry for three independent experiments are also shown below Western blots. Before coimmunoprecipitation, dHL-60 cells were exposed for 30 min to control buffer (HBSS^+/+^), 285 nM HxA_3_, 200 nM of the positive allosteric CB_2_R modulator EC21a, or both HxA_3_ and EC21a, as well as 100 nM *f*MLP as control (**C** and **D**). We observe an alternate molecular weight form of TRPV2 in coimmunoprecipitation as the predominant band. Representative Western blots and averaged band densities as means ± SEM for at least three independent experiments (*n* = 3). Statistical analysis performed by one-way ANOVA with Bonferroni correction for multiple comparisons. *P < 0.05; ns, *P* > 0.05.

Pull-downs of CB_2_R probed for TRPV2 demonstrated that these two proteins could associate. At 15 min post–HxA_3_ treatment, there is an increase in the amount of TRPV2 present in CB_2_R pull-down, even when cells were simultaneously treated with the positive allosteric modulator agonist EC21a (Fig. 6A). Notably, a slight but consistent increase in apparent molecular size was observed for TRPV2 in association with CB_2_R, consistent with posttranslational modifications associated with this channel trafficking to the plasma membrane (*32*, *33*). Thus, pull-downs showing association with immunoprecipitated CB_2_R may highlight events occurring at the plasma membrane and not at intracellular sites. In the reverse format, pull-down of TRPV2 resulted in a moderate level of CB_2_R coprecipitated under all conditions but less than input (Fig. 6B). In this pull-down format, no change in the apparent molecular size of CB_2_R was observed. Although EC21a treatment decreased overall TRPV2 protein levels in the presence of HxA_3_ compared to HxA_3_ alone (Fig. 4, C and E), it had no appreciable effect on the amount of TRPV2 coimmunoprecipitated with CB_2_R (Fig. 6A), indicating that HxA_3_ may act to stabilize TRPV2-CB_2_R complexes already at the membrane. As a verification of HxA_3_ specificity, the amount of TRPV2 present in CB_2_R pull-downs (Fig. 6C) and CB_2_R in TRPV2 pull-downs (Fig. 6D) was not affected by *f*MLP.

The observed TRPV2-CB_2_R interaction was specific. Pull-down of CB_1_R failed to coprecipitate TRPV2 in untreated or HxA_3_-treated dHL-60 cells (fig. S8A). Pull-down of TRPV2 did not appear to coprecipitate CB_1_R (fig. S8B), with or without 30-min exposure to HxA_3_. TRPV1, also known as the capsaicin receptor (*34*), was pulled down from dHL-60 cells that were untreated or exposed to 100 nM capsaicin for 30 min and the lysates probed for CB_2_R. While treatment of dHL-60 cells with capsaicin resulted in an apparent increase in total TRPV1 protein levels, no association was observed between pulled-down TRPV1 and probed CB_2_R (fig. S8C). Similarly, pull-down of CB_2_R demonstrated no physical association with TRPV1 in dHL-60 cells (fig. S8D). Together, these results show that TRPV2 and CB_2_R directly and specifically interact, suggesting that their roles in the HxA_3_-mediated neutrophil migration may be entangled.

**Fig. 7.**
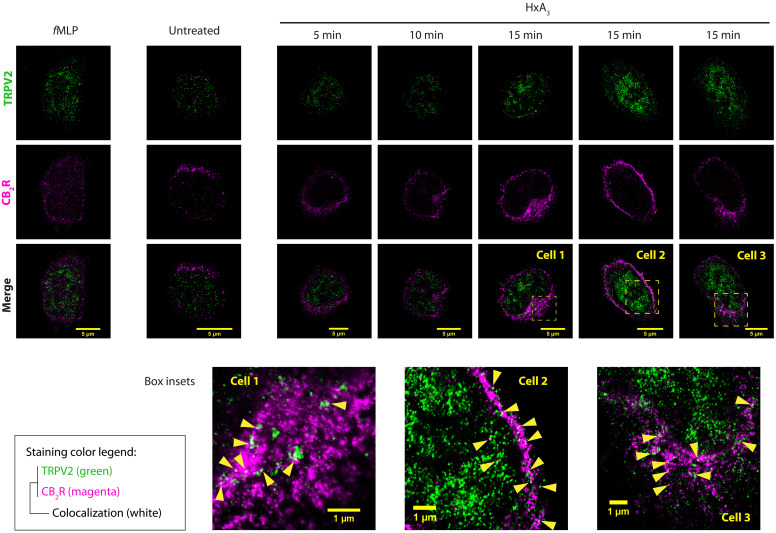
Proximity of TRPV2 and CB2R is consistent with colocalization by STED microscopy. Representative STED images (single *z* planes, 20- to 40-nm resolution for colocalization) of human peripheral neutrophils demonstrating proximity of TRPV2 (green) and CB_2_R (magenta) and their colocalization (white; select instances highlighted by arrowheads on insets) after treatment with HxA_3_. Cells were treated for 15 min with 100 nM *f*MLP or left untreated and compared to treatment for 5, 10, or 15 min with 285 nM HxA_3_. Scale bars, 5 or 1 μm (box insets).

To better appreciate the proximity of TRPV2-CB_2_R interactions, we used STED (stimulated emission depletion) microscopy with a 20- to 30-nm resolution (*35*). As in dHL-60 cells, CB_2_R was observed at the cell surface, and TRPV2 was present at intracellular sites when examined in freshly isolated human peripheral neutrophils (Fig. 7). Exposure to HxA_3_ resulted in TRPV2 movement to the cell surface where it colocalized with CB_2_R. Treatment with *f*MLP failed to induce TRPV2 migration to the cell surface and directional TRPV2-CB_2_R colocalizations. These results suggest that the cellular redistribution of TRPV2 and cell surface TRPV2-CB_2_R colocalizations induced by HxA_3_ in dHL-60 cells were repeated in peripheral human neutrophils and that TRPV2-CB_2_R cell surface proximity was consistent with physical contact between these two proteins. Together, these data suggest that HxA_3_-induced organization of TRPV2-CB_2_R complexes forms at the leading edge of human neutrophils and that these structures are dynamic.

### TRPV2 and CB_2_R show differential receptor cycling fates

**Fig. 8.**
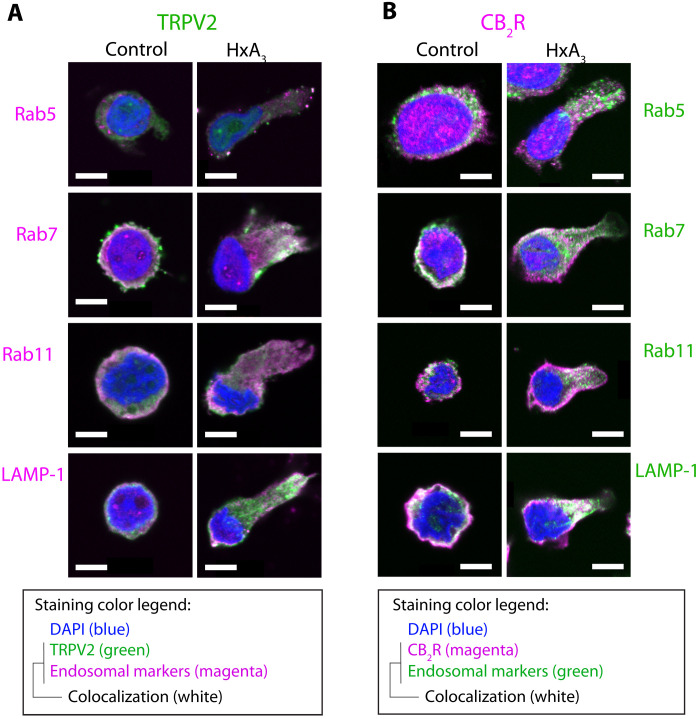
Chemoattractant-induced intracellular vesicular trafficking of TRPV2 and CB_2_R shows distinctive recycling paths. Representative fluorescent microscopic images (single *z* planes) of dHL-60 cells showing distribution of (**A**) TRPV2 (green) or (**B**) CB_2_R (magenta) and vesicular markers for early endosome (Rab5), late endosome (Rab7), recycling endosome (Rab11), or lysosome (LAMP-1) after 15 min of exposure to HBSS^+/+^ buffer (control) or 285 nM HxA_3_. Colocalization with endosomal markers appears white with general direction of cell migration to right/top right. Composite images use DAPI (blue) for nuclear staining. Scale bars, 5 μm.

That disparate localizations of TRPV2 or CB_2_R were observed in dHL-60 cells before and after exposure to HxA_3_ (Fig. 5) suggests that intracellular trafficking events for these proteins may be divergent and reflect distinct cellular fates. To test this possibility, we examined TRPV2- or CB_2_R-containing vesicle populations in dHL-60 cells before and after a 15 min of exposure to HxA_3_ or *f*MLP for markers of early (Rab5), late (Rab7), and recycling (Rab11) endosomes, as well as lysosomes demonstrated by lysosomal-associated membrane protein 1 (LAMP-1) (Fig. 8). Initially, TRPV2 was located primarily at intracellular sites, with minimal colocalizations with Rab5, and moderate colocalization with Rab7 and LAMP-1. Colocalization with Rab11 was less consistent than with Rab7 or LAMP-1. Exposure to HxA_3_ resulted in TRPV2^+^ vesicles being distributed throughout the pseudopod and at the plasma membrane with infrequent colocalizations with Rab5 throughout the cell. There was a notable increase in TRPV2 colocalizations with Rab7, Rab11, and LAMP-1. Colocalizations with Rab7 were present in the pseudopod body, while LAMP-1 colocalizations were focused to its leading edge. Treatment of dHL-60 cells for 15 min with *f*MLP (fig. S9) showed infrequent colocalizations of TRPV2 with Rab5 or Rab7. The extent of TRPV2 colocalizing with Rab11 and LAMP-1 was less following *f*MLP treatment compared to HxA_3_ treatment.

CB_2_R was distributed mostly at the cell surface in untreated dHL-60 cells, demonstrating less colocalizations with Rab5 intracellularly, but extensive colocalizations with Rab7, Rab11, and LAMP-1 at or near the plasma membrane. At 15 min post–HxA_3_ treatment, CB_2_R showed intracellular colocalizations with Rab5, primarily within the body of the pseudopod. Rab7-CB_2_R colocalizations, however, were observed near the plasma membrane throughout the cell except for the leading area of the pseudopod. Rab11-CB_2_R colocalizations were concentrated within the pseudopod closer to the nucleus than the leading edge. CB_2_R–LAMP-1 colocalizations remained prominent, being distributed throughout the pseudopod, but not at its leading edge as was observed for TRPV2–LAMP-1. In comparison, treatment of dHL-60 cells with *f*MLP showed extensive colocalization with Rab5 throughout the pseudopod and minimal colocalization with Rab7 and Rab11, while colocalization with LAMP-1 in both uropod and pseudopod regions was distinct from that observed following HxA_3_ treatment.

These studies showed that TRPV2 in the pseudopod region of dHL-60 cells migrating in response to HxA_3_ was associated with Rab7, consistent with this channel being present in signaling endosomes (*36*). While Rab11-invovled recycling of TRPV2 appears to be occurring in the uropod area in response to HxA_3_, lysosomal (identified by LAMP-1) down-regulation of the channel appeared to be occurring in the pseudopod. Following HxA_3_ exposure, the pattern of CB_2_R colocalized extensively with LAMP-1 and moderately with Rab7 and Rab11 along the body, but not the leading edge, of the pseudopod was consistent with membrane delivery and recycling along with down-regulation. CB_2_R colocalizations with Rab5 and Rab11 observed in the pseudopod were consistent with studies showing this cannabinoid receptor to undergo Rab5-mediated internalization and recycling to the plasma membrane in this region of the cell via a Rab11-dependent pathway (*37*).

## DISCUSSION

Recognition of a pathogenic challenge in the intestinal lumen is met with an innate immune response that includes the coordinated delivery of neutrophils to the site of infection, orchestrated largely by signaling from intestinal epithelial cells (*38*). Critically, neutrophils in the lamina propria must remain inactivated until they reach the intestinal lumen to minimize host tissue damage before pathogen engagement. We previously identified the eicosanoid HxA_3_, effluxed by MRP2, to function as a pure neutrophil chemoattractant that is distinct from other chemotactic agents such as *f*MLP, which incite neutrophil activation in addition to chemotaxis. The apical positioning of MRP2 on epithelial cells allows HxA_3_ efflux to establish a chemotactic gradient capable of drawing neutrophils from the lamina propria into the intestinal lumen. Although HxA_3_ does not activate neutrophils, unchecked HxA_3_ efflux appears to predispose patients to an inflammatory state, as evidenced by the relative increase of MRP2 and decrease in P-gp documented in patients with IBD (*39*–*42*) and significant increases in HxA_3_ relative to eCBs in inflamed versus noninflamed tissues observed in intestinal biopsies of patients with IBD. Thus, an understanding how HxA_3_ could draw neutrophils to the intestinal lumen and the interplay of this process with P-gp expression could provide new insights into treating inflammatory conditions.

Here, we identify the cation channel TRPV2 to be a critical element in the chemotaxis of neutrophils in response to HxA_3_ in a process that involves translocation of this channel to the leading edge of pseudopod structures. At the cell surface, TRPV2 physically interacts with CB_2_R to produce a complex that can differentially regulate the chemotactic actions of HxA_3_ and suppress the actions of eCBs. By establishing a sensing structure responsive to an HxA_3_ gradient and eCBs, CB_2_R-TRPV2 complexes at the plasma membrane appear to “prime” neutrophils in the lamina propria where they could be held in a state of migration stasis until experiencing a sufficient HxA_3_ gradient. We also show that TRPV2-CB_2_R complex removal from the cell surface involves distinct events of CB_2_R recycling and TRPV2 degradation, potentially maintaining the eCB-associated suppressor element presence while refreshing the HxA_3_-responsive element. Along with the labile nature of HxA_3_, this system could provide a mechanism for not only maintaining directional fidelity to changes in HxA_3_ gradients but also rapidly resolving neutrophil migration when the stimulus for epithelial cell HxA_3_ production is eliminated.

**Fig. 9.**
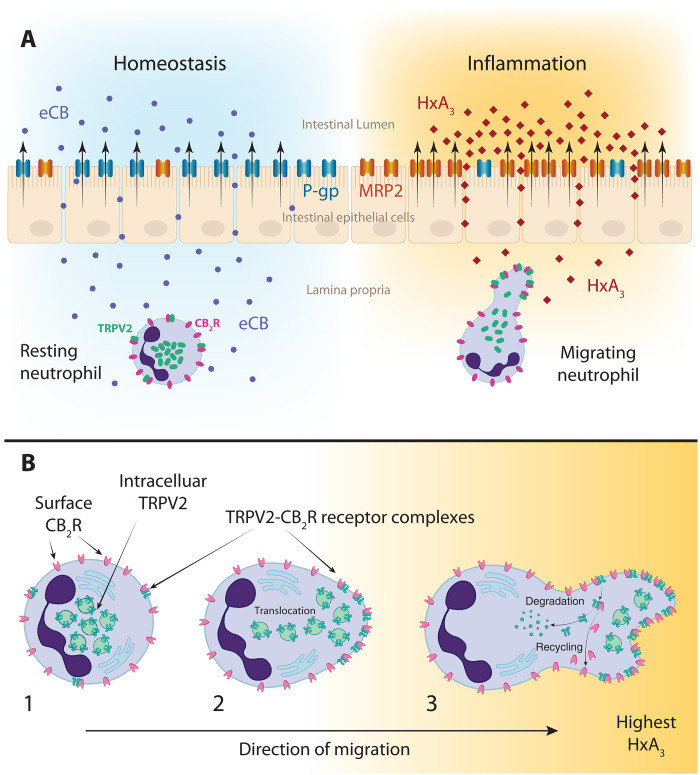
TRPV2-CB_2_R interaction add nuance to the current model HxA_3_-induced directional neutrophil chemotaxis. (**A**) Neutrophils respond to small-molecule signals produced by intestinal epithelial cells during homeostasis and acute inflammation. P-gp and MRP2 are two of the efflux transporters present at the apical surface of intestinal epithelial cells. During homeostasis, P-gp expression and its efflux of eCBs set conditions within the lamina propria where neutrophils are held in “ready stasis” by eCB activation of CB_2_R. In acute intestinal inflammation, P-gp is down-regulated, while MRP2 is up-regulated, establishing a chemotactic gradient of HxA_3_ across the intestinal epithelium. Neutrophils within the lamina propria sense and respond to HxA_3_ via TRPV2 activation. (**B**) HxA_3_ induces directional migration of neutrophils coordinated by changes in intracellular trafficking of both TRPV2 and CB_2_R. (1) Resting neutrophils express CB_2_R throughout their plasma membranes with infrequent TRPV2-CB_2_R receptor complexes. (2) HxA_3_ activation/signaling through plasma membrane TRPV2-CB2R receptor complexes triggers translocation of intracellular TRPV2 to the plasma membrane, creating more TRPV2-CB_2_R receptor complexes at the pseudopod, directing HxA_3_-gradient driven neutrophil migration. (3) Selective degradation and recycling of TRPV2 and CB_2_R maintain directional fidelity of neutrophil migration.

We now propose an integrated, closed-loop system of bioactive lipids, efflux pumps, and cell surface receptors that regulate neutrophil migration across polarized intestinal epithelial cells (Fig. 9) where intestinal epithelial cells function as a sensor to discriminate between commensal and pathogenic bacteria and communicate with neutrophils in the lamina propria via eCBs and HxA_3_. The dependence of TRPV2 activity on the physical presence of and contact with CB_2_R provides a mechanism for the duality of neutrophil “priming” in the lamina propria without activation in preparation to rapidly respond to migratory signaling from HxA_3_. CB_2_R activation using EC21a followed by HxA_3_ suppressed TRPV2 protein levels to a lesser degree than simultaneous treatment with EC21a and HxA_3_, implying a mechanism by which activated CB_2_R readies neutrophils for HxA_3_-mediated migration through stabilization of CB_2_R-TRPV2 sensing complexes. TRPV2 may also be directly affected by eCBs such as arachidonoyl ethanolamine (AEA) and 2-AG, as well as the cannabinoid Δ^9^-tetrahydrocannabinol, which can bind at its vanilloid binding pocket (VBP) (*23*, *43*). Structural studies indicate that VBP ligand binding can widen but does not fully open the pore (*44*), sensitizing it to activation by 2-APB (*45*) possibly through CB_2_R conformational changes (*44*). Thus, TRPV2 priming may depend on relative concentration of eCBs versus HxA_3_, its presence at the membrane, and other dynamic physiologic regulatory mechanisms, which balance out inhibitory versus stimulatory signaling cascades via the TRPV2-CB_2_R complex.

These studies further define the mechanism used by HxA_3_ to direct neutrophil migration and further clarify how this is distinct from the actions of other chemotactic agents, such as *f*MLP, that simultaneously induces neutrophil activation. One limitation of the present study is a lack of understanding of how neutrophils transition from pure chemotaxis induced by HxA_3_, to avoid premature activation, to responding to *f*MLP upon reaching an infective agent. Our studies are also limited by a lack of demonstration of leading-edge TRPV2-CB_2_R polarization in neutrophils within the environment of the lamina propria during human intestinal infection. While our studies have shown TRPV-CB_2_R complex formation at the leading edge of migrating cells, they are limited by a lack of complete understanding of the mechanisms that control the formation and function of this complex, issues to be addressed in future studies. We have shown TRPV2 to specifically interacted with CB_2_R and not CB_1_R, but it is unclear whether other G protein–coupled receptor (GPCR) proteins could affect the behavior or properties of the signaling system established by TRPV2-CB_2_R complexes. In this regard, CB_2_R can interact with the GPCR proteins GPR55 in a complex that can modulate CB_2_R-mediated responses (*46*). Thus, another limitation to the current studies is that it is currently unclear whether other GPCR proteins may play a role in the formation and function of TRPV2-CB_2_R complexes.

In sum, these results add to our understanding of mechanisms regulating the movement of neutrophils from peripheral blood to the luminal surface of the intestinal epithelium, a process critical for a potent innate immune response to pathogens but which also can become dysregulated in chronic inflammation. Pathogen sensing results in nuclear factor κB–regulated chemokines (e.g., IL-8) from intestinal epithelial cells that act on endothelial cells and locally induce neutrophil diapedesis. Neutrophils reaching the basal surface of intestinal epithelial cells in the lamina propria are then exposed to a mixture of lipids, including HxA_3_ and eCBs secreted from specific efflux pumps MRP2 and P-gp, respectively, that are expressed at the apical epithelial surface. Our studies suggest that eCBs can act through CB_2_R present on neutrophils in the lamina propria to prime processes involved in cell motility, placing them in a state poised for transepithelial migration. This priming action can appear to have both activating and inhibiting actions on the overall transmigration process (*47*), but it is the presence of an HxA_3_ concentration gradient acting on TRPV2 channels of eCB-primed neutrophils that drives directional transepithelial migration. This sequence outlines a potential scenario where neutrophils can leave the systemic circulation to reach a perceived pathogen in the intestinal lumen, being directed by a unique mechanism used by neutrophils where receptors that drive both inhibition and stimulation of cell migration form a leading-edge complex.

## MATERIALS AND METHODS

### Study design

This study sought to identify and characterize the neutrophil receptor directly responsible for HxA_3_-mediated neutrophil migration. We used dHL-60 cells, which originate from a human myeloid line but can be differentiated into a neutrophil phenotype for in vitro assays. Specific phenotype was confirmed through cell surface markers and neutrophil migration assays; key study assays were also replicated in freshly isolated human neutrophils. To identify the receptor directly activated by HxA_3_, we developed a receptor internalization and capture workflow (fig. S2 and described below) using two biotinylation steps. Captured internalized receptors were then identified by mass spectrometry (MS). Given prior knowledge of HxA_3_ inducing Ca^2+^ currents, we focused on cation channel candidates. We used specific inhibitors and siRNA knockdown to identify TRPV2 as specific for HxA_3_-induced neutrophil migration in Transwell-based assays. We next sought to characterize conditions that influenced expression and activity of TRPV2 using pharmacologic inhibitors of CB_2_R, which is known to oppose HxA_3_-mediated neutrophil migration, in combination with qPCR, semiquantitative Western blot, and siRNA knockdowns. Unexpectedly, CB_2_R was found to be required for TRPV2 functional activity. To characterize the nature of interaction between CB_2_R and TRPV2, we used immunofluorescent imaging and coimmunoprecipitation to show that they physically interact at the neutrophil plasma membrane and that they have different intracellular recycling fates after TRPV2 activation by HxA_3_. All experiments were repeated at least three times. Representative immunofluorescent images were chosen for figures.

### HxA_3_ purification and enrichment

We previously described the extraction and enrichment protocol and purity of HxA_3_ (*48*, *49*). Briefly, to induce HxA_3_ efflux from human epithelial cells, *Pseudomonas aeruginosa* strain PA01 was grown aerobically in LB broth overnight at 37°C. Cultures were washed once in HBSS^+/+^ and suspended at a concentration of 6 × 10^7^ bacteria/ml. H292 monolayers in 175-cm^2^ flasks were infected for 1 hour, washed with HBSS^+/+^, and then incubated in HBSS^+/+^ for 2 hours. Collected supernatants were captured by reversed phase chromatography on octadecylsilane (C_18_) columns (Supelco, Sigma-Aldrich), washed with water, and eluted with methanol. Samples were stored at −80°C, and the volume necessary for individual experiments was dried down and suspended in HBSS^+/+^ as needed. Each new batch of enriched HxA_3_ was quality tested before use in experiments and was generally used after a 1:4 to 1:8 dilution.

### dHL-60 cell culture and differentiation

The human promyelocytic leukemia (HL-60) cell line was obtained from the American Type Culture Collection (catalog number CCL-240). Cells were cultured in Iscove’s modified Dulbecco’s medium (1× IMDM; Gibco, Thermo Fisher Scientific, USA) supplemented with 10% heat-inactivated fetal bovine serum (FBS; Gibco, Thermo Fisher Scientific) and 1% penicillin-streptomycin (10,000 U/ml; Gibco, Thermo Fisher Scientific) in 175-cm^2^ flasks (Thermo Fisher Scientific, 178883) at 37°C and 5% CO_2_. HL-60 cells were passaged every 2 to 3 days and passaged were no more than 30 times.

HL-60 cell suspensions were differentiated into the neutrophil surrogate dHL-60 cells as previously described (*50*). Briefly, HL-60 cells were collected from the T175 flasks, counted, centrifuged at 125*g* for 5 min at room temperature, and resuspended in differentiation medium [1× IMDM supplemented with 10% of non–heat-inactivated FBS (Gibco, Thermo Fisher Scientific), 1% penicillin-streptomycin (10,000 U/ml; Gibco, Thermo Fisher Scientific), and 1.27% of dimethyl sulfoxide (Sigma-Aldrich)] at an approximate concentration of 1 × 10^5^ cells in 100 ml in a T175 cm^2^ flask (Thermo Fisher Scientific, 178883) or 200 ml in a 500-cm^2^ triple flask (Thermo Fisher Scientific, 132935). Cells were incubated at 37°C and 5% CO_2_ for 6 to 8 days without changing medium to fully differentiate into dHL-60. Phenotype was confirmed by flow cytometry (see below).

### Human neutrophil isolation

Fresh primary neutrophils were extracted from healthy human volunteers on the same morning that assays were performed. All donors were consented on the risks and benefits of providing research material by blood draw for each donation according to our institutional review board policies and protocol (UMass Chan Institutional review board approval no. MOD00005953). Signed consent forms were securely kept in locked storage by an authorized manager. No identifiable or nonidentifiable information about the donor was collected or associated with donated blood. Peripheral blood neutrophils from healthy human volunteers were isolated from acid citrate dextrose–anticoagulated peripheral blood by 2% gelatin sedimentation, as previously described (*51*). Red blood cells were removed by lysis in cold NH_4_Cl buffer, and neutrophils were washed with HBSS^−/−^ (without Ca^2+^ or Mg^2+^) and suspended to a final volume of 5 × 10^7^/ml for use in experiments.

### Flow cytometry confirmation of neutrophil-like phenotype of dHL-60 cells and characterization of isolated human neutrophils

Single-cell suspensions of dHL-60 or undifferentiated HL-60 cells were collected from T175 cell culture flasks and centrifuged at 125*g* for 5 min at 4°C. Cell density was adjusted to 0.5 × 10^6^ to 1 × 10^6^ cells/100 μl and resuspended in fluorescence-activated cell sorting (FACS) buffer [1× phosphate-buffered saline (PBS) and 1% bovine serum albumin (BSA)] in Eppendorf tubes. Freshly isolated human neutrophils were resuspended at 0.5 × 10^6^ to 1 × 10^6^ cells/100 μl in FACS buffer. Cell surface nonspecific Fc-binding sites were blocked with Fc receptor binding inhibitor polyclonal antibody (eBioscience, UK, 14-9161-73) diluted at 1:50 in FACS buffer for 30 min on ice. Cells were washed by centrifugation at 125*g* for 5 min at 4°C, and supernatants were discarded. Cells were labeled with 5 μg of anti-human CD11b–fluorescein isothiocyanate–conjugated monoclonal antibody (M1/70) (Invitrogen, UK, MA1-1008) in 100 μl of FACS buffer and incubated for 30 min on ice while protected from light with aluminum foil. Viability staining was performed with 1 μl of fixable viability dye eFluor 780 (eBioscience, 65-0865-14) in 100 μl of FACS buffer for 10 min on ice with anti-CD11b incubation. Cells were washed thrice by centrifugation (125*g* for 5 min at 4°C), resuspended in 200 μl of FACS buffer, and analyzed on the BD FACSCanto or Bio-Rad ZE5 Cell Analyzer. Dead cells were excluded (640-nm excitation and 775/50 emission filter), and the mean fluorescence intensity of CD11b (488-nm excitation and 525/35 emission filter) was measured.

### Migration assays

For migration assays using dHL-60, cell suspensions were collected by centrifugation at 125*g* for 5 min at 4°C. The cell concentration was adjusted to 1 × 10^6^ cells in 100 μl of HBSS^+/+^. Migration studies were performed in 96-well Transwell plates (5.0-μm pore size; Corning, USA). In-house purified HxA_3_ (see above), HxA_3_ methyl ester (Cayman Chemical, USA, 22151), 2-APB (Bio-Techne Ltd., UK, 1224), or *f*MLP (Cayman Chemical, USA, 21495) was added to the lower compartment at the indicated concentrations in a volume of 100 μl per well in HBSS^+/+^ as chemotactic agents to induce transmigration. To test agents/conditions that may disrupt HxA_3_-induced transmigration, dHL-60 were treated for 15 min with agents dissolved in HBSS^+/+^: tranilast (100 nM; Bio-Techne Ltd., UK, 1098), CB_2_R-positive allosteric modulator agonist EC21a (C2) (100 nM), the CB_2_R-selective orthosteric agonist LV-62 (*6*) (100 nM), dual orthosteric agonist FM-6b (150 nM) or dualsteric bitopic orthosteric/allosteric agonist FD22a (150 nM), AEA (Cayman Chemical, USA, CAS number 94421-68-8), palmitoyl ethanolamide (Cayman Chemical, USA, CAS number 544-31-0) O-AEA (Cayman Chemical, USA, CAS number 443129-35-9), α-linoleoyl ethanolamide (Cayman Chemical, USA, CAS number 68171-52-8), and 2-AG (Cayman Chemical, USA, CAS number 53847-30-6). EC21a, LV-62, FM-6b, and FD22a were provided by Professor Clementina Manera at Università di Pisa. Treated or control dHL-60 cells (1 × 10^6^ in 100 μl of HBSS^+/+^) were added to the upper chamber, placed in a 37°C incubator with 5% CO_2_ and allowed to migrate for 15 to 60 min.

For migration assays using human neutrophils, 6.5-mm Transwell filter plates (Corning, USA; 5-μm pore size) were coated with rat tail collagen (0.1 mg/ml; Sigma-Aldrich, catalog number C7661) and allowed to dry overnight. Enriched HxA_3_ (see above), 2-APB, or *f*MLP at the indicated concentrations was added to the lower compartment. Tranilast and EC21a, at the indicated concentrations, were added to both compartments. Neutrophils (2 × 10^6^ in 40 μl) were added to the top well, placed in a 37°C incubator with 5% CO_2_, and allowed to migrate for 2 hours.

To quantify transmigrated dHL-60 cells or human neutrophils, top wells were removed, and transmigrated cells in the bottom compartment were lysed with 1% Triton X-100 (Thermo Fisher Scientific, UK, 9002-93-1). Sodium citrate buffer (0.1 M, pH 4.2; Thermo Fisher Scientific, UK, 6132-04-3 and 68-04-2) was added, and an equal volume of 2,2′-azino-bis 3-ethylbenzothiazoline 6-sulfonic acid (Sigma-Aldrich, USA, 30931-67-0) was added to samples. Myeloperoxidase (MPO) activity was measured, and cell numbers were calculated by comparison with a standard curve, with data from individual experiments being normalized to 100% HxA_3_-driven migration. Statistical analysis was performed using GraphPad Prism.

### HxA_3_ receptor screen and identification

A total of 2 × 10^7^ dHL-60 cells were washed twice by centrifugation at 500*g* for 5 min using ice-cold HBSS^+/+^. Cell surface proteins were biotinylated according to the manufacturer’s instructions (Pierce, USA, 89881). Briefly, cells were incubated with sulfo–*N*-hydroxysuccinimide–SS–biotin solution at a concentration of 0.25 mg/ml in ice-cold HBSS^+/+^ with gentle rocking for 30 min at 4°C. Cells were then treated with a quenching solution for 5 min at 4°C and washed once in ice-cold HBSS^+/+^ before splitting into equal aliquots. Split cells were incubated at room temperature with HxA_3_ (50 μg/ml) or HBSS^+/+^ at 37°C for 5 min. To label noninternalized surface proteins, cells were centrifuged (500*g* for 5 min) and resuspending in streptavidin (Rockland, USA, S1000-01) solution at 58 μg/ml in ice-cold PBS with gentle rocking at 4°C for 20 min, followed by one wash of ice-cold tris-buffered saline (TBS). Cells were then resuspended thoroughly and lysed by addition of radioimmunoprecipitation assay (RIPA) lysis buffer with protease inhibitors on ice for 30 min with sonication every 15 min. Cell lysates were centrifuged at 10,000*g* at 4°C for 2 min. Separation of internalized proteins from the lysate was achieved by agarose bead capture of biotinylated proteins, which were not streptavidin labeled in the prior step. Briefly, cell lysates were applied to Pierce NeutrAvidin Agarose (Thermo Fisher Scientific, 29200) bead columns according to the manufacturer’s instructions and incubated at room temperature with gentle rotation for 30 min. Unbound material was removed by spinning the column at 1000*g* for 1 min and washing the column three times with TBS.

Tandem mass tag (TMT) labeling and high-pH reversed-phase chromatography in preparation for nano–liquid chromatography (LC)–MS was completed by University of Bristol Proteomics Centre (Bristol, UK) using protocols and analysis methods described previously (*52*) with some modifications. The enriched membrane proteins were reduced (10 mM tris(2-carboxyethyl)phosphine, 55°C for 1 hour), alkylated (18.75 mM iodoacetamide, room temperature for 30 min), and then digested from the beads with trypsin (2.5 μg of trypsin, 37°C, overnight). The resulting peptides were then labeled with TMT six-plex reagents according to the manufacturer’s protocol (Thermo Fisher Scientific), and the labeled samples were pooled and desalted using a Sep-Pak cartridge according to the manufacturer’s instructions (Waters, Milford, MA, USA). Eluate from the Sep-Pak cartridge was evaporated to dryness and resuspended in buffer A [20 mM ammonium hydroxide (pH 10)] before fractionation by high-pH reversed-phase chromatography using an Ultimate 3000 LC system (Thermo Fisher Scientific). Briefly, the sample was loaded onto an XBridge BEH C_18_ column (130 Å, 3.5 μm, 2.1 mm by 150 mm; Waters, UK) in buffer A, and peptides were eluted with an increasing gradient of buffer B [20 mM ammonium hydroxide in acetonitrile (pH 10)] from 0 to 95% over 60 min. The resulting fractions were evaporated to dryness and resuspended in 1% formic acid before analysis by nano–LC-MS/MS using an Orbitrap Fusion Tribrid mass spectrometer (Thermo Fisher Scientific).

High-pH reversed-phase fractions were further fractionated using an Ultimate 3000 nano-LC system in line with an Orbitrap Fusion Tribrid mass spectrometer (Thermo Fisher Scientific). Briefly, peptides in 1% (v/v) formic acid were injected onto an Acclaim PepMap C18 nano-trap column (Thermo Fisher Scientific). After washing with 0.5% (v/v) acetonitrile and 0.1% (v/v) formic acid, peptides were resolved on a 250 mm–by–75 μm Acclaim PepMap C18 reversed-phase analytical column (Thermo Fisher Scientific) over a 150-min organic gradient with a flow rate of 300 nl/min. Solvent A was 0.1% formic acid, and solvent B was aqueous 80% acetonitrile in 0.1% formic acid. Peptides were ionized by nanoelectrospray ionization at 2.0 kV using a stainless steel emitter with an internal diameter of 30 μm (Thermo Fisher Scientific) and a capillary temperature of 275°C.

All spectra were acquired using an Orbitrap Fusion Tribrid mass spectrometer controlled by Xcalibur 2.1 software (Thermo Fisher Scientific) and operated in data-dependent acquisition mode using an SPS-MS3 workflow. Initial fourier transformed mass spectra were collected at a resolution of 120,000, with an automatic gain control (AGC) target of 200,000 and a maximum injection time of 50 ms. Precursors were filtered with an intensity threshold of 5000, according to charge state (to include charge states 2 to 7) and with monoisotopic peak determination set to peptide. Previously interrogated precursors were excluded using a dynamic window [60 s, ±10 parts per million (ppm)]. The MS2 precursors were isolated with a quadrupole isolation window of 1.2 mass/charge ratio. Ion trap mass spectrometry stage 2 spectra were collected with an AGC target of 10,000, a maximum injection time of 70 ms, and a collision-induced dissociation collision energy of 35%.

For FTMS3 analysis, the Orbitrap was operated at resolution of 50,000 with an AGC target of 50,000 and a maximum injection time of 105 ms. Precursors were fragmented by high energy collision dissociation at a normalized collision energy of 60% to ensure maximal TMT reporter ion yield. Synchronous Precursor Selection (SPS) was enabled to include up to 10 MS2 fragment ions in the FTMS3 scan.

Raw data files were processed and quantified using Proteome Discoverer software v2.1 (Thermo Fisher Scientific) and searched against the Swiss-Prot Human database (downloaded July 2023; 20,334 sequences) and the Common Repository of FBS proteins (274 sequences) using the SEQUEST HT algorithm (*53*). Peptide precursor mass tolerance was set at 10 ppm, and MS/MS tolerance was set at 0.6 Da. Search criteria included oxidation of methionine (+15.995 Da), acetylation of the protein N terminus (+42.011 Da), and methionine loss plus acetylation of the protein N terminus (−89.03 Da) as variable modifications and carbamidomethylation of cysteine (+57.021 Da) as a fixed modification. Searches were performed with full tryptic digestion, and a maximum of two missed cleavages were allowed. The reverse database search option was enabled, and all data were filtered to satisfy a false discovery rate of 5%.

### Western blot

Protein was obtained from dHL-60 cell lysates (1 × 10^6^ cells) through the incubation of cells with RIPA lysis buffer [25 mM tris-HCl (pH 7.6), 150 mM NaCl, 1% NP-40, 1% sodium deoxycholate, and 0.1% SDS (Thermo Fisher Scientific, UK, 10017003] plus 1× protease inhibitor cocktail (cOmplete, Mini, EDTA-free, Roche, 11836170001) on ice for 30 min. Cellular debris was pelleted by centrifugation 12,000*g* for 15 min at 4°C. Supernatants (lysates) were collected and kept at −80°C or used directly for the determination of protein concentration and normalization using BCA protein assay kit (Thermo Fisher Scientific, UK, 23227). Proteins were separated by SDS–polyacrylamide gel electrophoresis (PAGE) (20 μl loaded into 4 to 12% or 3 to 8% polyacrylamide gels, NuPAGE, Invitrogen, Thermo Fisher Scientific, USA) under reducing conditions. Proteins were transferred in polyvinylidene difluoride blotting membrane (8.4 cm by 7 cm; Thermo Fisher Scientific, USA, 22860) for 1.5 hours at 150 V. Membranes were blocked with 4% BSA in TBS supplemented with 0.1% Tween 20 (TBST; Sigma-Aldrich, USA) for 1 hour. Membranes were probed with primary antibodies (1:1000; diluted in TBST plus BSA 4%) and left overnight at 4°C. Membranes were washed thrice for 10 min with TBST buffer (20 mM tris, 0.15 M NaCl, and 0.1% Tween 20) and probed with secondary antibodies (1:5000; diluted in TBST plus BSA 4%) for 1 hour (see table S1 below for antibody sources and catalog numbers). Membranes were then imaged using the LI-COR fluorescence Odyssey CLx Imaging system. ImageJ software (https://imagej.net/) was used to quantify protein bands. The measurement started with making a frame in the first lane and used the same frame for all the protein bands across the other lanes that gave a background measurement in the row. Integrated density values for targeted proteins and loading control (β-actin) were calculated by subtracting nearby background. The corrected density of the protein of interest was then divided by corrected density of the loading control to obtain normalized intensity for the band of interest. Mean values of experimental groups were divided by control group values to determine fold change, with values represented in a graph bar (GraphPad Prism software).

### dHL-60 siRNA treatment

After differentiation (see above), dHL-60 cell suspensions were collected from T175 cm^2^ culture flasks seeded at 3 × 10^5^ cells per well in 12-well cell culture plates (Corning, USA, 3513) in a volume of 200 μl per well. Sense and antisense siRNA oligos (see table S2 below) were diluted to a concentration of 750 ng (100 nM) in 200 μl of IMDM without serum. HiPerFect Transfection Reagent (6 μl; QIAGEN, 301705) was added to the diluted siRNAs and mixed by vortexing to produce complexes that were applied to cells and incubated for 5 to 10 min at room temperature before incubation under normal growth conditions for 6 hours at 37°C and 5% CO_2_. After this period, 400 μl of culture medium containing serum and antibiotics was added to the cells and incubated for 24 hours.

### Real-time qPCR

RNA extraction was performed using the PureLink RNA Mini Kit (Life Technologies, 12183018A) following the manufacturer’s instructions. Briefly, dHL-60 cells were collected, counted, and centrifuged at 125*g* for 5 min at room temperature, before being adjusted to a concentration 5 × 10^6^ cells/ml. Cells were then lysed with 0.6 ml of lysis buffer and 2-mercaptoethanol (1%) for 30 min at room temperature. Cellular debris was pelleted by centrifugation at 12,000*g* for 15 min at 4°C. Supernatants were collected, and 70% ethanol was added before vortexing to disperse any visible precipitate.

#### RNA binding, washing, and elution

Up to 700 μl of the samples were added to a spin cartridge with a collection tube and centrifuged at 12,000*g* for 15 s at room temperature. The samples were washed three times with 700 μl of wash buffer I, and the flow-through was discarded. The spin cartridge was placed into a new collection tube, and 500 μl of wash buffer II with ethanol was added into the spin cartridge and centrifuged at 12,000*g* for 15 s. The RNA was eluted with 50 to 100 μl of ribonuclease (RNase)–free water by centrifuging at 12,000*g* for 2 min at room temperature. The RNA start point concentration was measured by NanoDrop and adjusted to 100 ng with the 260/280-nm absorbance ratio between 1.8 and 2.1, confirming purity.

#### Quantitative polymerase chain reaction

Analysis was performed on the StepOne Real-Time PCR instrument (Applied Biosystems) using the SYBR Green I detection kit (QuantiNova SYBR Green, QIAGEN, UK, 208154) according to the manufacturer’s instructions. Locked nucleic acid–enhanced primers for human TRPV2 were ordered from QIAGEN (QIAGEN GeneGlobe ID: SBH0500613, product name: HS_TRPV2_1744427, main target: ENST00000338560, catalog number 249990). Briefly, 2× SYBR Green reverse transcription (RT)–PCR master mix (11 μl) was incubated with 20× primer mix [0.5 μM; dissolved in 10 mM tris-HCl and 1 mM EDTA (pH 8.0) buffer], QuantiNova ROX reference dye (1 μl), QuantiNova SYBR Green RT-Mix, which contains HotStarRT-Script Reverse Transcriptase for heat-mediated activation of the RT step, an RNase inhibitor (0.2 μl) and RNA template (variable concentration) plus RNase-free water (variable) in a total reaction volume of 20 μl in a 96-well PCR microplate (Axygen, Corning). The cycling conditions were 50°C for 10 min, 95°C for 2 min (holding stage), 95°C for 5 s, 60°C for 10 s (cycling stage), and 40 cycles. Data were analyzed using the StepOne software version 2.3 (Applied Biosystems).

### Confocal microscopy

dHL-60 cells were collected from T175 flasks, counted, centrifuged at 125*g* for 5 min at room temperature and adjusted a concentration of 5 × 10^5^ cells in 300 μl of HBSS^+/+^ for each condition. Cells in this volume were then seeded onto single chambers of poly-l-lysine (Sigma-Aldrich, P4707) coated μ-Slide eight-well glass-bottom chamber slides (ibidi, catalog number 80807/Thistle Scientific, catalog number IN-80807). Cells were allowed to adhere for 1 to 2 hours at 37°C with 5% CO_2_ before any pharmacologic treatments. Cells were then fixed with paraformaldehyde (4%; Sigma-Aldrich, P6148) for 20 min at room temperature, washed with PBS, blocked for 30 min (PBS supplemented with 10% FBS and 1% Triton X-100, Sigma-Aldrich), and then treated with primary antibodies diluted at 1:100 in dilution buffer [PBS plus 1% BSA (Sigma-Aldrich) and 0.01% sodium aside (Sigma-Aldrich)] for 1 hour at room temperature. After washing twice with wash buffer (PBS with BSA 1% BSA and Triton X-100 0.1%, Sigma-Aldrich), samples were treated with secondary antibodies (1:100 in dilution buffer) and then buffer washed twice. 4′,6-Diamidino-2-phenylindole (DAPI; Sigma-Aldrich, D9542-1MG) was added to each well for 20 min before one buffer wash and covering with anti-fade fluorescence mounting medium (aqueous) (Thermo Fisher Scientific, AB104135) (see table S1 below for antibodies and catalog numbers). Cells were imaged using confocal microscopy (Zeiss LSM 880 confocal, Zeiss); images were analyzed with ZEN microscopy software (Zeiss).

### STED microscopy

Human neutrophils were isolated as described above. Cells were seeded at 7.5 × 10^5^ cells per well onto 24-well glass-like polymer bottom tissue culture treated imaging plates (Cellvis, catalog number P24-1.5P) and allowed to adhere for 30 min at 37°C with 5% CO_2_. Cells were then treated with HxA_3_ or *f*MLP for specified times at 37°C with 5% CO_2_, as described above, before fixation and staining for confocal imaging, as described above, except that STED-specific secondary antibodies were used (see table S1 below). Samples were then imaged using the STED imaging module on the Leica Microsystems Stellaris 8 Microscope with white light laser, Leica Microsystems HC PL APO CS2 100×/1.40 oil objective (PN 506378) and 775-nm STED depletion laser, controlled by LAS X 4.8.1.29271. The 775-nm depletion laser was set at 60% power, with excitation at 590 nm set to 5% power and excitation at 653 nm set to 1.2% power. STED images with 590-nm laser settings were captured using TauSTED using HyD S3 detector, and 653-nm laser settings were captured using TauGating method with a gate of 0.3 to 8 ns on the HyD S3 detector. Confocal images for each channel were captured simultaneously for reference. All imaging settings were constant for all samples.

### Coimmunoprecipitation

dHL-60 cell suspensions were collected at 1 × 10^7^ cell density and incubated with 1 ml of RIPA lysis buffer (Thermo Fisher Scientific, UK, 10017003) plus 1× protease inhibitor cocktail (complete, Mini, EDTA-free, Roche, 11836170001) per sample condition on ice for 30 min. Cell debris was pelleted by centrifugation 12,000*g* for 15 min at 4°C. Lysate protein concentration was determined using BCA protein assay kit (Thermo Fisher Scientific, 23227). Primary antibodies (1 to 2 μg/ml) were added to lysates and incubated using a rocker platform at 4°C for 1 hour. After primary antibody incubation, Protein A/G Plus Agarose beads (20 μl/ml; Santa Cruz Biotechnology, SC-2003) were added to lysates before overnight incubation at 4°C using a rocker platform. Immunoprecipitants were collected by centrifugation at 2,500*g* for 5 min at 4°C. Pellets were washed four times with 1 ml of RIPA buffer, and the supernatants were discarded, with the pellets being resuspended in 40 μl of 1× electrophoresis sample buffer ( lithium dodecyl sulfate sample buffer, nonreducing 4×, Thermo Fisher Scientific, 84788). Immunocomplexes were separated by SDS-PAGE (20 μl loaded onto 4 to 12% or 3 to 8% polyacrylamide gels, NuPAGE, Invitrogen, Thermo Fisher Scientific, USA), and Western blot was performed as described above. Bands were quantified as described above (see table S1 below for antibodies and catalog numbers).

### Materials used in this study

Primary and secondary antibodies for Western Blots, coimmunoprecipitation, and immunofluorescence assays can be found in table S1. Sequences and catalog numbers for siRNAs can be found in table S2.

### Statistical analyses

GraphPad Prism software (version 10.4.2) was used to perform all statistical analyses. All experiments were repeated at least three independent times with sample/technical replicates. Statistical analysis for comparisons between two samples were performed by one-way analysis of variance (ANOVA) with Bonferroni correction for multiple comparisons. Data are displayed as means ± SD.

## References

[1] M. Phillipson, P. Kubes, The healing power of neutrophils. Trends Immunol. 40, 635–647 (2019). 31160208 10.1016/j.it.2019.05.001

[2] T. Kucharzik, J. T. Hudson III, A. Lugering, J. A. Abbas, M. Bettini, J. G. Lake, M. E. Evans, T. R. Ziegler, D. Merlin, J. L. Madara, I. R. Williams, Acute induction of human IL-8 production by intestinal epithelium triggers neutrophil infiltration without mucosal injury. Gut 54, 1565–1572 (2005). 15987794 10.1136/gut.2004.061168 PMC1774758

[3] R. L. Szabady, B. A. McCormick, Control of neutrophil inflammation at mucosal surfaces by secreted epithelial products. Front. Immunol. 4, 220 (2013). 23914188 10.3389/fimmu.2013.00220 PMC3728559

[4] C. H. T. Hall, E. L. Campbell, S. P. Colgan, Neutrophils as components of mucosal homeostasis. Cell. Mol. Gastroenterol. Hepatol. 4, 329–337 (2017). 28884136 10.1016/j.jcmgh.2017.07.001 PMC5581871

[5] R. J. Mrsny, A. T. Gewirtz, D. Siccardi, T. Savidge, B. P. Hurley, J. L. Madara, B. A. McCormick, Identification of hepoxilin A3 in inflammatory events: A required role in neutrophil migration across intestinal epithelia. Proc. Natl. Acad. Sci. U.S.A. 101, 7421–7426 (2004). 15123795 10.1073/pnas.0400832101 PMC409934

[6] M. Pazos, D. Siccardi, K. L. Mumy, J. D. Bien, S. Louie, H. N. Shi, K. Gronert, R. J. Mrsny, B. A. McCormick, Multidrug resistance-associated transporter 2 regulates mucosal inflammation by facilitating the synthesis of hepoxilin A3. J. Immunol. 181, 8044–8052 (2008). 19017997 10.4049/jimmunol.181.11.8044 PMC2596662

[7] R. L. Szabady, C. Louissaint, A. Lubben, B. Xie, S. Reeksting, C. Tuohy, Z. Demma, S. E. Foley, C. S. Faherty, A. Llanos-Chea, A. J. Olive, R. J. Mrsny, B. A. McCormick, Intestinal P-glycoprotein exports endocannabinoids to prevent inflammation and maintain homeostasis. J. Clin. Invest. 128, 4044–4056 (2018). 30102254 10.1172/JCI96817 PMC6118593

[8] S. E. Foley, C. Tuohy, M. Dunford, M. J. Grey, H. De Luca, C. Cawley, R. L. Szabady, A. Maldonado-Contreras, J. M. Houghton, D. V. Ward, R. J. Mrsny, B. A. McCormick, Gut microbiota regulation of P-glycoprotein in the intestinal epithelium in maintenance of homeostasis. Microbiome 9, 183 (2021). 34493329 10.1186/s40168-021-01137-3 PMC8425172

[9] E. S. Onaivi, G. Chaudhuri, A. S. Abaci, M. Parker, D. H. Manier, P. R. Martin, J. R. Hubbard, Expression of cannabinoid receptors and their gene transcripts in human blood cells. Prog. Neuropsychopharmacol. Biol. Psychiatry 23, 1063–1077 (1999). 10621950 10.1016/s0278-5846(99)00052-4

[10] D. Reynaud, P. M. Demin, C. R. Pace-Asciak, Coupling of hepoxilin A3-specific binding with calcium-mobilizing actions in human neutrophils. Agents Actions Suppl. 45, 291–296 (1995). 7717192 10.1007/978-3-0348-7346-8_39

[11] L. Mills, D. Reynaud, C. R. Pace-Asciak, Hepoxilin-evoked intracellular reorganization of calcium in human neutrophils: A confocal microscopy study. Exp. Cell Res. 230, 337–341 (1997). 9024792 10.1006/excr.1996.3425

[12] T. Maritzen, H. Schachtner, D. F. Legler, On the move: Endocytic trafficking in cell migration. Cell. Mol. Life Sci. 72, 2119–2134 (2015). 25681867 10.1007/s00018-015-1855-9 PMC11113590

[13] F. Merino-Casallo, M. J. Gomez-Benito, S. Hervas-Raluy, J. M. Garcia-Aznar, Unravelling cell migration: Defining movement from the cell surface. Cell Adh. Migr. 16, 25–64 (2022). 35499121 10.1080/19336918.2022.2055520 PMC9067518

[14] S. J. Collins, The HL-60 promyelocytic leukemia cell line: Proliferation, differentiation, and cellular oncogene expression. Blood 70, 1233–1244 (1987). 3311197

[15] I. Heiner, J. Eisfeld, A. Luckhoff, Role and regulation of TRP channels in neutrophil granulocytes. Cell Calcium 33, 533–540 (2003). 12765698 10.1016/s0143-4160(03)00058-7

[16] X. Cao, Y. Li, Y. Luo, T. Chu, H. Yang, J. Wen, Y. Liu, Y. Zhao, M. Herrmann, Transient receptor potential melastatin 2 regulates neutrophil extracellular traps formation and delays resolution of neutrophil-driven sterile inflammation. J. Inflamm. 20, 7 (2023). 10.1186/s12950-023-00334-1 PMC9945693 36810113

[17] M. Zhang, N. Kang, X. Yu, X. Zhang, Q. Duan, X. Ma, Q. Zhao, Z. Wang, X. Wang, Y. Liu, Y. Zhang, C. Zhu, R. Gao, X. Min, C. Li, J. Jin, Q. Cao, R. Liu, X. Bai, H. Yang, L. Zhao, J. Liu, H. Chen, Y. Zhang, W. Liu, W. Zheng, TNF inhibitors target a mevalonate metabolite/TRPM2/calcium signaling axis in neutrophils to dampen vasculitis in Behcet’s disease. Nat. Commun. 15, 9261 (2024). 39461948 10.1038/s41467-024-53528-3 PMC11513106

[18] F. H. Robledo-Avila, J. D. Ruiz-Rosado, K. L. Brockman, S. Partida-Sanchez, The TRPM2 ion channel regulates inflammatory functions of neutrophils during *Listeria monocytogenes* infection. Front. Immunol. 11, 97 (2020). 32117251 10.3389/fimmu.2020.00097 PMC7010865

[19] T. Shimizu, K. Kanai, Y. Kyo, K. Asano, T. Hisamitsu, H. Suzaki, Effect of tranilast on matrix metalloproteinase production from neutrophils in-vitro. J. Pharm. Pharmacol. 58, 91–99 (2006). 16393468 10.1211/jpp.58.1.0011

[20] V. Juvin, A. Penna, J. Chemin, Y. L. Lin, F. A. Rassendren, Pharmacological characterization and molecular determinants of the activation of transient receptor potential V2 channel orthologs by 2-aminoethoxydiphenyl borate. Mol. Pharmacol. 72, 1258–1268 (2007). 17673572 10.1124/mol.107.037044

[21] F. Gado, K. A. Mohamed, S. Meini, R. Ferrisi, S. Bertini, M. Digiacomo, F. D’Andrea, L. A. Stevenson, R. B. Laprairie, R. G. Pertwee, C. Manera, Variously substituted 2-oxopyridine derivatives: Extending the structure-activity relationships for allosteric modulation of the cannabinoid CB2 receptor. Eur. J. Med. Chem. 211, 113116 (2021). 33360803 10.1016/j.ejmech.2020.113116

[22] V. Lucchesi, D. P. Hurst, D. M. Shore, S. Bertini, B. M. Ehrmann, M. Allara, L. Lawrence, A. Ligresti, F. Minutolo, G. Saccomanni, H. Sharir, M. Macchia, V. Di Marzo, M. E. Abood, P. H. Reggio, C. Manera, CB2-selective cannabinoid receptor ligands: Synthesis, pharmacological evaluation, and molecular modeling investigation of 1,8-naphthyridin-2(1*H*)-one-3-carboxamides. J. Med. Chem. 57, 8777–8791 (2014). 25272206 10.1021/jm500807e PMC4234427

[23] L. Zhang, C. Simonsen, L. Zimova, K. Wang, L. Moparthi, R. Gaudet, M. Ekoff, G. Nilsson, U. A. Hellmich, V. Vlachova, P. Gourdon, P. M. Zygmunt, Cannabinoid non-cannabidiol site modulation of TRPV2 structure and function. Nat. Commun. 13, 7483 (2022). 36470868 10.1038/s41467-022-35163-y PMC9722916

[24] M. U. Ehrengruber, D. A. Deranleau, T. D. Coates, Shape oscillations of human neutrophil leukocytes: Characterization and relationship to cell motility. J. Exp. Biol. 199, 741–747 (1996). 8788084 10.1242/jeb.199.4.741

[25] L. E. Hind, W. J. Vincent, A. Huttenlocher, Leading from the back: The role of the uropod in neutrophil polarization and migration. Dev. Cell 38, 161–169 (2016). 27459068 10.1016/j.devcel.2016.06.031 PMC4982870

[26] L. P. Cramer, Forming the cell rear first: Breaking cell symmetry to trigger directed cell migration. Nat. Cell Biol. 12, 628–632 (2010). 20596043 10.1038/ncb0710-628

[27] K. Harada, T. Kitaguchi, T. Kamiya, K. H. Aung, K. Nakamura, K. Ohta, T. Tsuboi, Lysophosphatidylinositol-induced activation of the cation channel TRPV2 triggers glucagon-like peptide-1 secretion in enteroendocrine L cells. J. Biol. Chem. 292, 10855–10864 (2017). 28533434 10.1074/jbc.M117.788653 PMC5491772

[28] E. Hisanaga, M. Nagasawa, K. Ueki, R. N. Kulkarni, M. Mori, I. Kojima, Regulation of calcium-permeable TRPV2 channel by insulin in pancreatic β-cells. Diabetes 58, 174–184 (2009). 18984736 10.2337/db08-0862 PMC2606868

[29] M. Nagasawa, I. Kojima, Translocation of calcium-permeable TRPV2 channel to the podosome: Its role in the regulation of podosome assembly. Cell Calcium 51, 186–193 (2012). 22226146 10.1016/j.ceca.2011.12.012

[30] M. Nagasawa, I. Kojima, Translocation of TRPV2 channel induced by focal administration of mechanical stress. Physiol. Rep. 3, (2015). 10.14814/phy2.12296 PMC4393204 25677550

[31] M. Nagasawa, Y. Nakagawa, S. Tanaka, I. Kojima, Chemotactic peptide fMetLeuPhe induces translocation of the TRPV2 channel in macrophages. J. Cell. Physiol. 210, 692–702 (2007). 17154364 10.1002/jcp.20883

[32] J. C. Barnhill, A. J. Stokes, M. Koblan-Huberson, L. M. Shimoda, A. Muraguchi, C. N. Adra, H. Turner, RGA protein associates with a TRPV ion channel during biosynthesis and trafficking. J. Cell. Biochem. 91, 808–820 (2004). 14991772 10.1002/jcb.10775

[33] R. Jahnel, O. Bender, L. M. Munter, M. Dreger, C. Gillen, F. Hucho, Dual expression of mouse and rat VRL-1 in the dorsal root ganglion derived cell line F-11 and biochemical analysis of VRL-1 after heterologous expression. Eur. J. Biochem. 270, 4264–4271 (2003). 14622291 10.1046/j.1432-1033.2003.03811.x

[34] C. L. Baker, J. J. McDougall, The cannabinomimetic arachidonyl-2-chloroethylamide (ACEA) acts on capsaicin-sensitive TRPV1 receptors but not cannabinoid receptors in rat joints. Br. J. Pharmacol. 142, 1361–1367 (2004). 15277316 10.1038/sj.bjp.0705902 PMC1575203

[35] G. Vicidomini, P. Bianchini, A. Diaspro, STED super-resolved microscopy. Nat. Methods 15, 173–182 (2018). 29377014 10.1038/nmeth.4593

[36] M. R. Cohen, W. M. Johnson, J. M. Pilat, J. Kiselar, A. DeFrancesco-Lisowitz, R. E. Zigmond, V. Y. Moiseenkova-Bell, Nerve growth factor regulates transient receptor potential vanilloid 2 via extracellular signal-regulated kinase signaling to enhance neurite outgrowth in developing neurons. Mol. Cell. Biol. 35, 4238–4252 (2015). 26416880 10.1128/MCB.00549-15 PMC4648816

[37] H. Stenmark, Rab GTPases as coordinators of vesicle traffic. Nat. Rev. Mol. Cell Biol. 10, 513–525 (2009). 19603039 10.1038/nrm2728

[38] J. Pott, M. Hornef, Innate immune signalling at the intestinal epithelium in homeostasis and disease. EMBO Rep. 13, 684–698 (2012). 22801555 10.1038/embor.2012.96 PMC3410395

[39] S. Ardizzone, G. Maconi, V. Bianchi, A. Russo, E. Colombo, A. Cassinotti, C. Penati, M. L. Tenchini, G. Bianchi Porro, Multidrug resistance 1 gene polymorphism and susceptibility to inflammatory bowel disease. Inflamm. Bowel Dis. 13, 516–523 (2007). 17260353 10.1002/ibd.20108

[40] H. Blokzijl, S. Vander Borght, L. I. Bok, L. Libbrecht, M. Geuken, F. A. van den Heuvel, G. Dijkstra, T. A. Roskams, H. Moshage, P. L. Jansen, K. N. Faber, Decreased P-glycoprotein (P-gp/MDR1) expression in inflamed human intestinal epithelium is independent of PXR protein levels. Inflamm. Bowel Dis. 13, 710–720 (2007). 17262809 10.1002/ibd.20088

[41] M. Brinar, S. Cukovic-Cavka, N. Bozina, K. G. Ravic, P. Markos, A. Ladic, M. Cota, Z. Krznaric, B. Vucelic, MDR1 polymorphisms are associated with inflammatory bowel disease in a cohort of Croatian IBD patients. BMC Gastroenterol. 13, 57 (2013). 23537364 10.1186/1471-230X-13-57 PMC3616873

[42] J. J. Zhao, D. Wang, H. Yao, D. W. Sun, H. Y. Li, CTLA-4 and MDR1 polymorphisms increase the risk for ulcerative colitis: A meta-analysis. World J. Gastroenterol. 21, 10025–10040 (2015). 26379408 10.3748/wjg.v21.i34.10025 PMC4566373

[43] C. Muller, P. Morales, P. H. Reggio, Cannabinoid ligands targeting TRP channels. Front. Mol. Neurosci. 11, 487 (2018). 30697147 10.3389/fnmol.2018.00487 PMC6340993

[44] M. V. Yelshanskaya, A. I. Sobolevsky, Ligand-binding sites in vanilloid-subtype TRP channels. Front. Pharmacol. 13, 900623 (2022). 35652046 10.3389/fphar.2022.900623 PMC9149226

[45] A. Gochman, X. F. Tan, C. Bae, H. Chen, K. J. Swartz, A. Jara-Oseguera, Cannabidiol sensitizes TRPV2 channels to activation by 2-APB. eLife 12, (2023). 10.7554/eLife.86166 PMC10195083 37199723

[46] N. A. Balenga, E. Aflaki, J. Kargl, W. Platzer, R. Schroder, S. Blattermann, E. Kostenis, A. J. Brown, A. Heinemann, M. Waldhoer, GPR55 regulates cannabinoid 2 receptor-mediated responses in human neutrophils. Cell Res. 21, 1452–1469 (2011). 21467997 10.1038/cr.2011.60 PMC3132458

[47] A. M. Miller, N. Stella, CB2 receptor-mediated migration of immune cells: It can go either way. Br. J. Pharmacol. 153, 299–308 (2008). 17982478 10.1038/sj.bjp.0707523 PMC2219538

[48] B. P. Hurley, A. Sin, B. A. McCormick, Adhesion molecules involved in hepoxilin A3-mediated neutrophil transepithelial migration. Clin. Exp. Immunol. 151, 297–305 (2008). 18005361 10.1111/j.1365-2249.2007.03551.x PMC2276941

[49] K. L. Mumy, J. D. Bien, M. A. Pazos, K. Gronert, B. P. Hurley, B. A. McCormick, Distinct isoforms of phospholipase A2 mediate the ability of *Salmonella enterica* serotype typhimurium and Shigella flexneri to induce the transepithelial migration of neutrophils. Infect. Immun. 76, 3614–3627 (2008). 18505810 10.1128/IAI.00407-08 PMC2493202

[50] G. D. Birnie, The HL60 cell line: A model system for studying human myeloid cell differentiation. Br. J. Cancer Suppl. 9, 41–45 (1988). 3076064 PMC2149104

[51] B. P. Hurley, D. Siccardi, R. J. Mrsny, B. A. McCormick, Polymorphonuclear cell transmigration induced by *Pseudomonas aeruginosa* requires the eicosanoid hepoxilin A3. J. Immunol. 173, 5712–5720 (2004). 15494523 10.4049/jimmunol.173.9.5712

[52] I. Urrutia-Irazabal, J. R. Ault, F. Sobott, N. J. Savery, M. S. Dillingham, Analysis of the PcrA-RNA polymerase complex reveals a helicase interaction motif and a role for PcrA/UvrD helicase in the suppression of R-loops. eLife 10, (2021). 10.7554/eLife.68829 PMC8318588 34279225

[53] J. Shin, Y. Kwon, S. Lee, S. Na, E. Y. Hong, S. Ju, H. G. Jung, P. Kaushal, S. Shin, J. H. Back, S. Y. Choi, E. H. Kim, S. J. Lee, Y. E. Park, H. S. Ahn, Y. Ahn, M. H. Kabir, S. J. Park, W. S. Yang, J. Yeom, O. Y. Bang, C. W. Ha, J. W. Lee, U. B. Kang, H. J. Kim, K. S. Park, J. E. Lee, J. E. Lee, J. Y. Kim, K. P. Kim, Y. Kim, H. Hirano, E. C. Yi, J. Y. Cho, E. Paek, C. Lee, Common repository of FBS proteins (cRFP) to be added to a search database for mass spectrometric analysis of cell secretome. J. Proteome Res. 18, 3800–3806 (2019). 31475827 10.1021/acs.jproteome.9b00475

