# Supplementary material for: Transient receptor potential vanilloid 2 functions as a directional driver for hepoxilin A3–mediated neutrophil migration
Source: Sci Adv. 2026 Jul 31;12(31):eadz1986. doi: 10.1126/sciadv.adz1986 (PMC13426407; doi:10.1126/sciadv.adz1986)
Supplement: Supplementary file 1 — Figs. S1 to S9 Tables S1 and S2 [file sciadv.adz1986_sm.pdf]

Science Advances
AAAS

Supplementary Materials for

Transient receptor potential vanilloid 2 functions as a directional driver for hepoxilin A_3–mediated neutrophil migration

Claudia Feriotti *et al.*

Corresponding author: Randall Mrsny, Rjm37@bath.ac.uk

Sci. Adv. **12**, eadz1986 (2026)
DOI: 10.1126/sciadv.adz1986

This PDF file includes:

Figs. S1 to S9
Tables S1 and S2

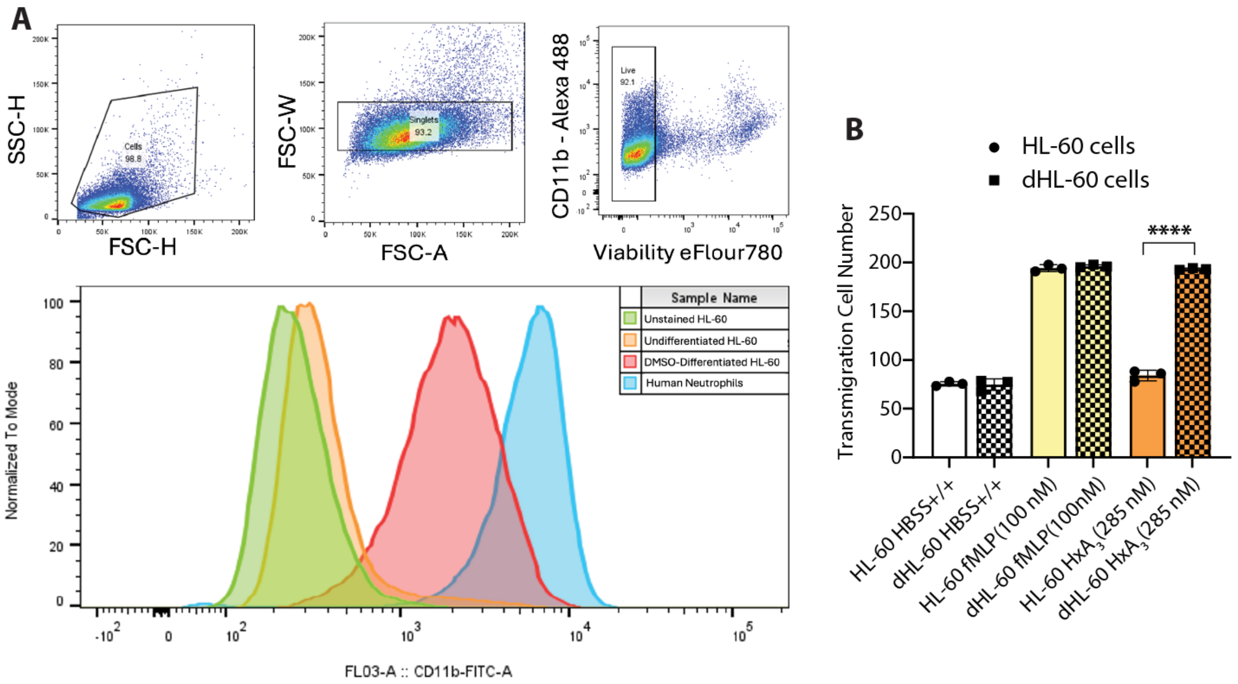

Figure S1. Differentiated HL-60 (dHL-60) cells show neutrophil phenotype as confirmed by flow cytometry and specific chemotactic migration. A) HL-60 differentiation was assessed by the leukocyte cell surface marker CD11b. HL-60 cells, before and after differentiation into dHL-60 by incubation in 1.25% dimethyl sulfoxide (DMSO) for 6 days along with freshly isolated primary human neutrophils, were labeled with anti-human CD11b and sorted by FACS using the gating strategy depicted (top panels). Shown is one replicate of at least three independent experiments. **B**) dHL-60 were tested for their ability to migrate in response to 100 nM bacterial chemotactic agent N-formyl-methionyl-leucyl-phenylalanine (fMLP) or 285 nM hepoxilin A_3 (HxA_3) versus the control buffer addition ($HBSS^{+/+}$) and undifferentiated HL-60. N = 3 independent experiments (three biologic replicates, each with at least three technical replicates) Statistical analysis performed by one-way ANOVA with Bonferroni correction for multiple comparisons. ****$p < 0.0001$.

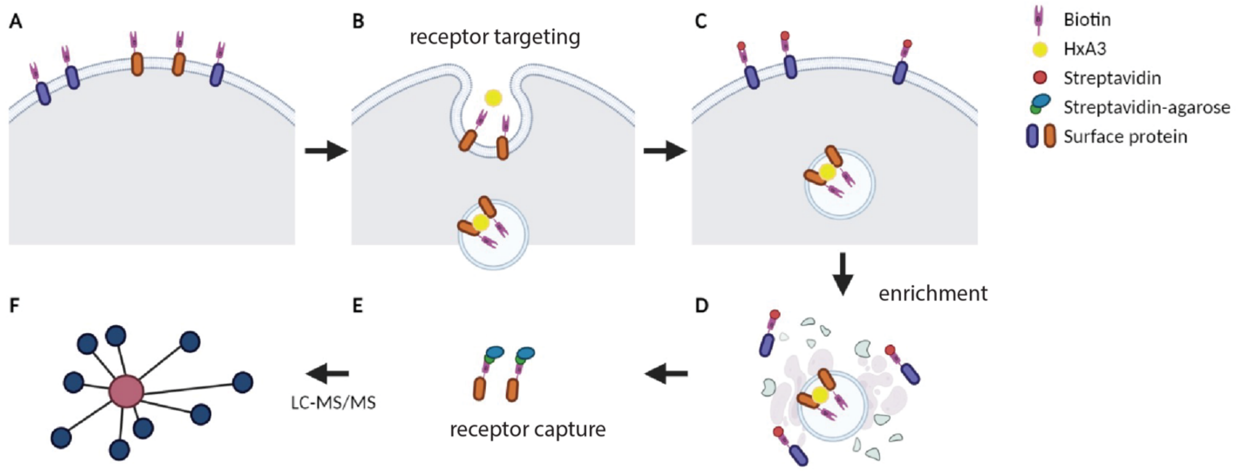

Figure S2. An enrichment/capture/analysis assay of cell surface proteins identified internalized receptors following hepoxilin A_3 (HxA_3) treatment. A) Sulfo-NHS-biotin treatment at 4°C for 30 min was used to label all cell surface proteins. **B**) Following washing at 4°C, cells were incubated with HxA_3 or control ($HBSS^{+/+}$) at 37°C for 5 min to allow receptor internalization. **C**) Remaining surface proteins are labelled with streptavidin, prior to washing and cell lysis at 4°C (**D**). Cells are fragmented to produced vesicles containing biotin-labelled, streptavidin-bound surface proteins and internalized biotin-labelled proteins. **E**) Internalized biotinylated proteins are captured using streptavidin-agarose magnetic beads, digested with trypsin, and (**F**) identified using LC-MS/MS.

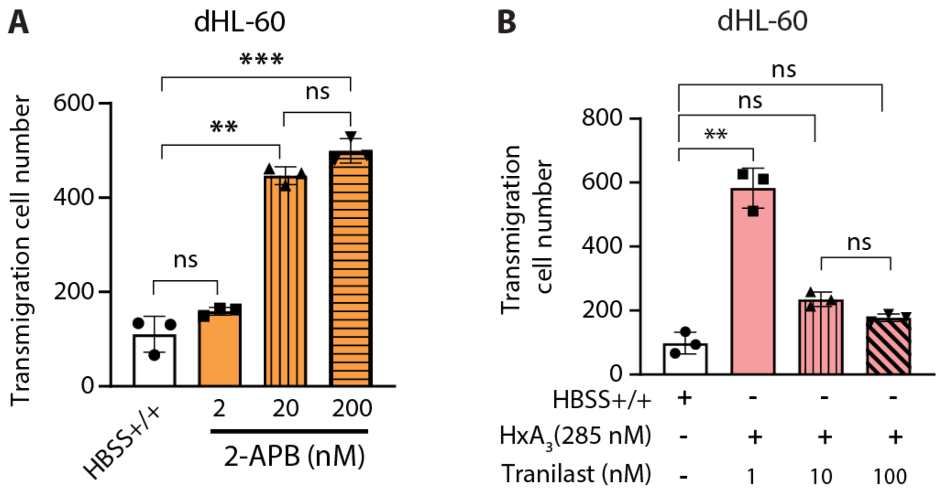

Figure S3. dHL-60 migrate in response to TRPV2 targeted agonism/antagonism. Dose-response curves for effects of **A**) the non-specific TRPV channel activator 2-aminoethoxydiphenyl borate (2-APB) on the *in vitro* migration of differentiated HL-60 (dHL-60) cells. **B**) Migration of dHL-60 in response to 285 nM hepoxilin A_3 (HxA_3) with various doses of TRPV2 channel blocker tranilast. Migration studies for dHL-60 cells were performed for 30 min at 37°C using Transwell® filters. Data are shown as mean ± SEM for at least three independent experiments (N=3) with at least three technical replicates each. Statistical analysis performed by one-way ANOVA with Bonferroni correction for multiple comparisons. ****$p < 0.0001$, ***$p < 0.001$, **$p < 0.01$, *$p < 0.05$, ns = $p > 0.05$.

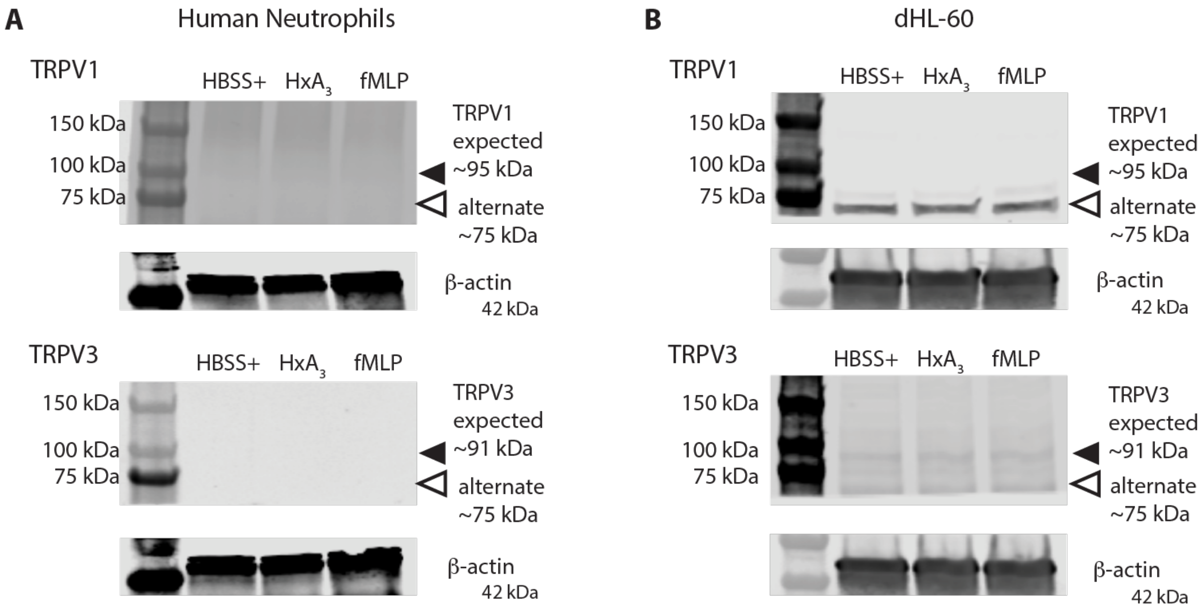

Figure S4. Total cellular TRPV1 and TRPV3 protein levels expression in human neutrophils are minimal. Western blot analysis of TRPV1 and TRPV3 expression levels in **A)** primary human neutrophils or **B)** HL-60 cells differentiated into a neutrophil-like phenotype (dHL-60) following exposure to control buffer (HBSS$^{+/+}$), 285 nM hepoxilin A_3 (HxA_3) or 100 nM N-formyl-methionyl-leucyl-phenylalanine (fMLP). Commonly reported alternate molecular weight forms are detected for TPRV1 and TPRV3 in dHL-60.

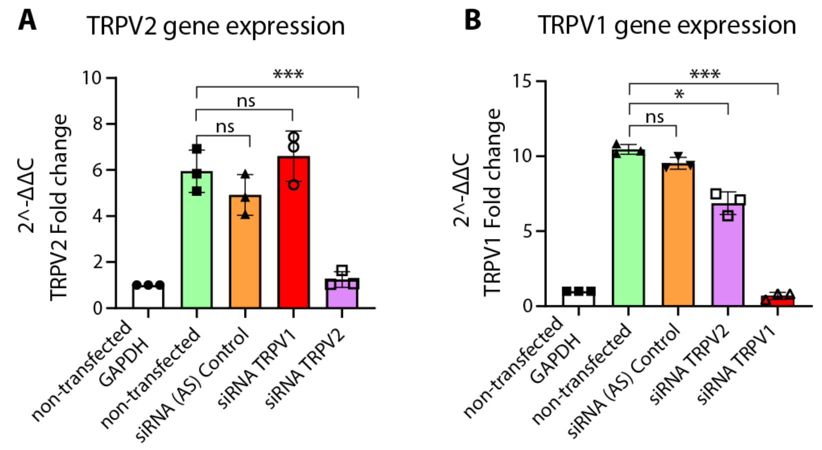

Figure S5. siRNA knockdown TRPV2 and TRPV1 gene expression in dHL-60 were confirmed by qPCR. Quantitative PCR of **A**) TRPV2 or **B**) TRPV2 gene expression in non-transfected (control) dHL-60 cells, or dHL-60 cells transfected with a negative control siRNA, or siRNA sequences designed to be specific for TRPV1 or TRPV2. GAPDH levels in non-transfected dHL-60 cells provided an internal control for comparison. Data are shown as mean ± SEM for at least three independent experiments (N=3). Statistical analysis performed by one-way ANOVA with Bonferroni correction for multiple comparisons. ****$p < 0.0001$, ***$p < 0.001$, **$p < 0.01$, *$p < 0.05$, ns = $p > 0.05$.

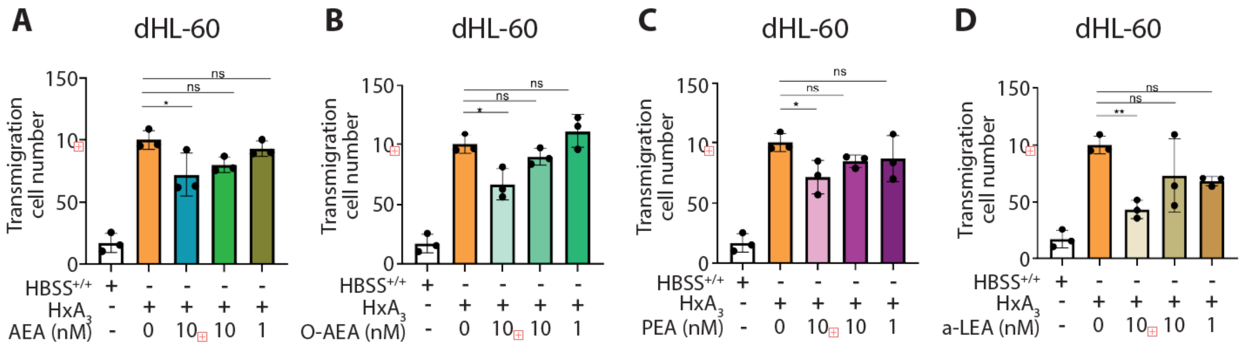

Figure S6. CB_2R activators variably affect HxA_3 – induced dHL-60 migration in a dose dependent manner. Migration of dHL-60 cells in response to hepoxilin A_3 (HxA_3) and in the presence of varying doses of **A**) arachidonoyl ethanolamine (AEA), **B**) O-arachidonoyl ethanolamine (O-AEA), **C**) palmitoyl ethanolamide (PEA), **D**) alpha-linoleoyl ethanolamide (a-LEA) were tested. Migration studies were performed for 30 min at 37°C using Transwell® filters. Data are shown as mean ± SEM for at least three independent experiments (N=3), each with at least three technical replicates. Statistical analysis performed by one-way ANOVA with Bonferroni correction for multiple comparisons: ****$p < 0.0001$, ***$p < 0.001$, **$p < 0.01$, *$p < 0.05$, ns = $p > 0.05$.

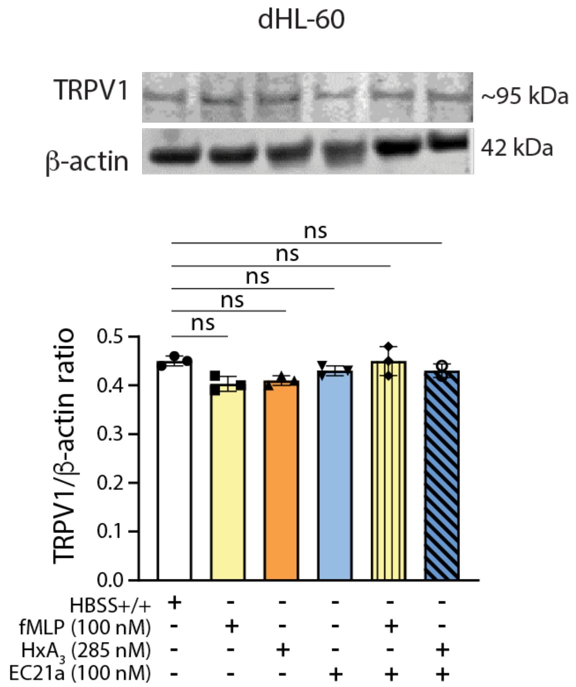

Figure S7. Total cellular TRPV1 protein levels in dHL-60 cells is not affected by HxA_3 or EC21a. Western blot of TRPV1 expression in dHL-60 cells following exposure to control buffer ($HBSS^{+/+}$), or combinations of 100 nM N-formyl-methionyl-leucyl-phenylalanine fMLP or 285 nM HxA_3 and 100 nM positive CB_2R allosteric modulator EC21a. Representative Western blot and averaged band densities showing mean ± SEM for at least three independent experiments (N=3). Statistical analysis performed by one-way ANOVA with Bonferroni correction for multiple comparisons. ****$p < 0.0001$, ***$p < 0.001$, **$p < 0.01$, *$p < 0.05$, ns = $p > 0.05$.

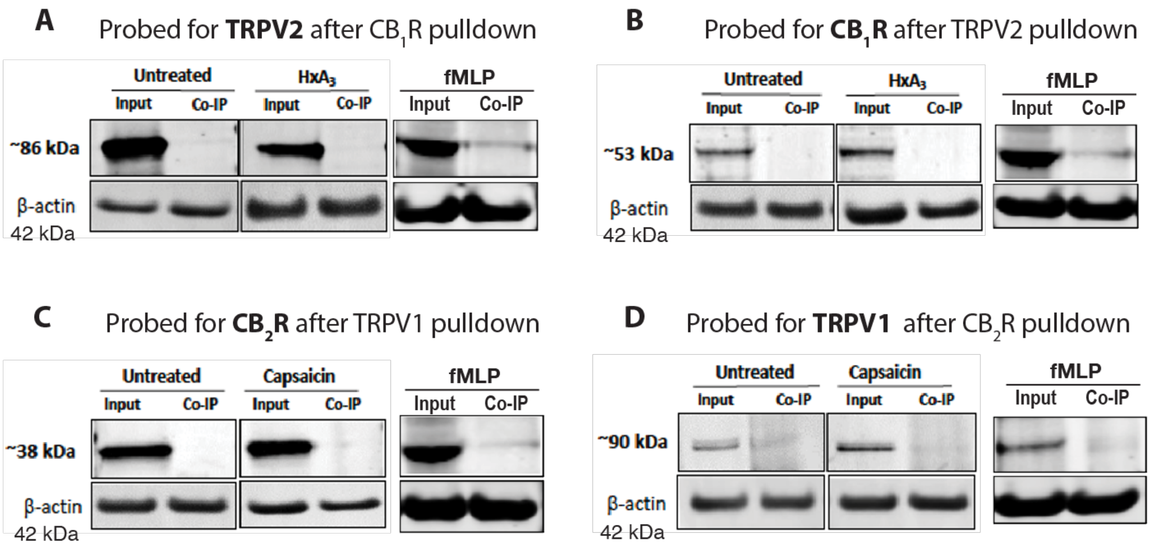

Figure S8. Controls using TRPV1 and CB_1R confirm TRPV2-CB_2R interaction specificity
To examine specificity of the TRPV2/CB_2R interaction, Co-IP analyses as in figure 6 were performed where dHL-60 cells were **A**) immunoprecipitated for CB_1R and probed for TRPV2, **B**) immunoprecipitated for TRPV2 and probed for CB_1R, **C**) immunoprecipitated for TRPV1 after stimulation with capsaicin and probed for CB_2R, or **D**) immunoprecipitated for CB_2R and probed for TRPV1. Representative Western blots.

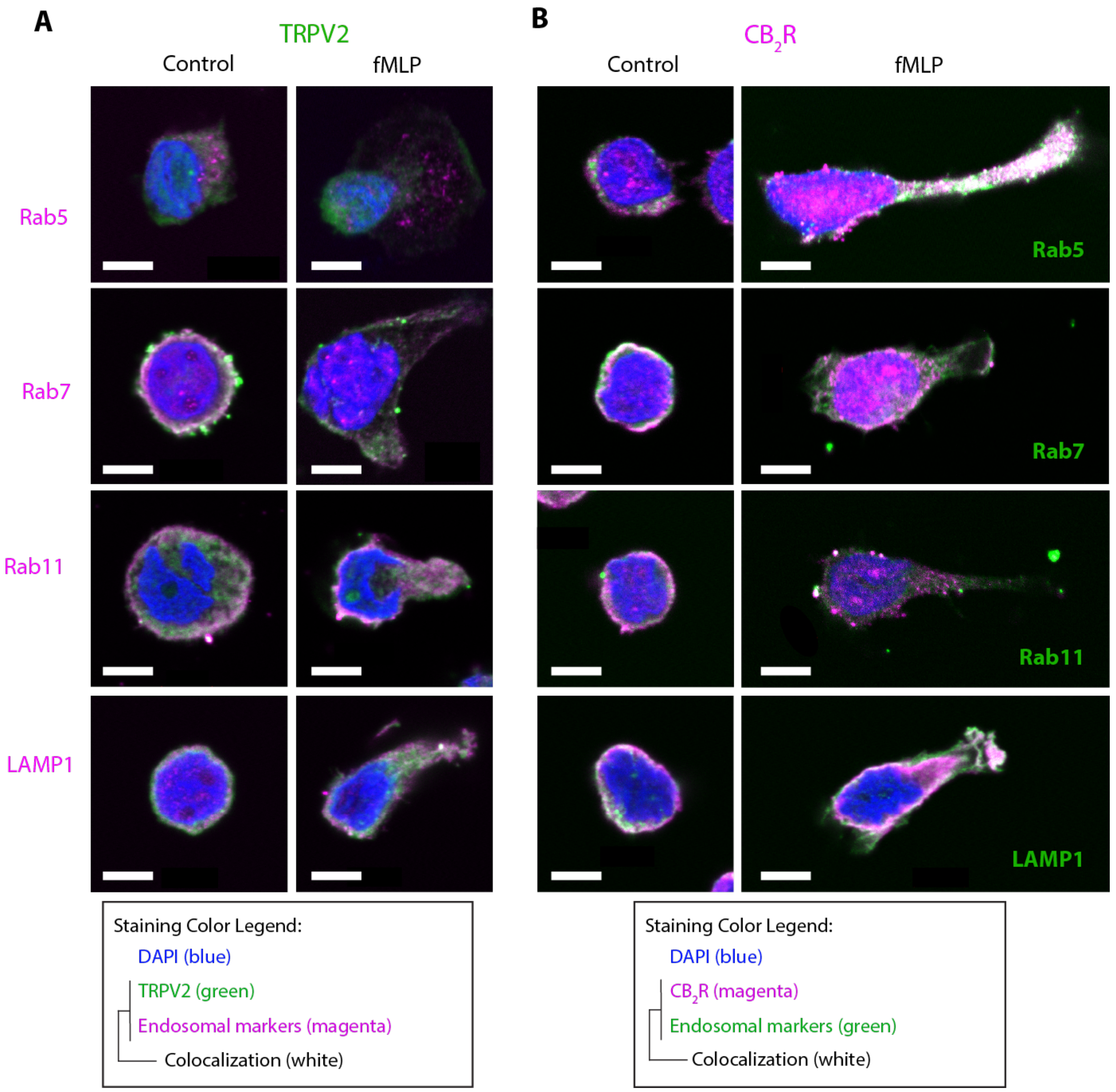

Figure S9. Intracellular vesicular trafficking of TRPV2 and CB_2R following exposure to N-formyl-methionyl-leucyl-phenylalanine (fMLP) show differential trafficking. Representative fluorescent microscopic images (single Z-planes) of dHL-60 cells showing the distribution of TRPV2 (green, left panels) or CB_2R (magenta, right panels) and versicular markers for early endosome (Rab5), late endosome (Rab7), recycling endosome (Rab11), or lysosome (LAMP-1) after 15 min of exposure to $HBSS^{+/+}$ buffer (control) or 100 nM fMLP. Co-localization of TRPV2 or CB_2R with endosomal markers appear white. Composite images use DAPI (blue) for nucleus staining. All scale bars are 5 μm.

Table S1: antibodies used in study

Antibody/Product	Catalog number	Source	Use
anti-human CD11b-FITC conjugated monoclonal Antibody (M1/70)	MA1-1008	Invitrogen	FC
TRPV2 rabbit polyclonal antibody	OSR00190W	Invitrogen	WB
human TRPV2 rabbit polyclonal antibody	PA5-27555	Invitrogen	WB, IMF
TRPV2 rabbit polyclonal antibody	15991-1-AP	Proteintech	WB
TRPV2 mouse monoclonal antibody (clone 2F11H3)	68563-1-Ig	Proteintech	CO:IP
TRPV1 Polyclonal Antibody	PA1-748	Invitrogen	WB
TRPV1 rabbit polyclonal antibody	PA5-34498	Invitrogen	CO:IP
TRPV3 mouse monoclonal antibody (Clone: S15-4)	MAB6666	Abnova	WB
human CNR2 mouse monoclonal antibody	H00001269-M01	Abnova	IMF
Cannabinoid R2/CB2/CNR2 Antibody (3C7) mouse monoclonal antibody	H00001269-M01	Novus Biologicals	WB
Cannabinoid receptor 2 rabbit polyclonal antibody	29371-1-AP	Proteintech	CO:IP
CNR1 mouse monoclonal antibody (Clone: 2F9)	H00001268-M01	Abnova	CO:IP
CNR1 rabbit polyclonal antibody	17978-1-AP	Proteintech	CO:IP
Sodium Potassium ATPase Recombinant Rabbit Monoclonal Antibody (ST0533)	MA5-32184	Invitrogen	WB
anti-b-actin Goat antibody	AB0145-200	SICGEN	WB
anti-human Rab5a mouse monoclonal antibody (2E8B11)	14-9711-82	eBioscience™	IMF
anti-human Rab7a goat polyclonal antibody	AB0033	SICGEN Antibodies	IMF
anti-human Rab11a rabbit polyclonal antibody	AB0034	SICGEN Antibodies	IMF
anti-human CD107a (LAMP-1) mouse monoclonal antibody (JJ0940)	MA5-32491	Invitrogen	IMF
**Secondary Antibodies**			
Donkey anti-rabbit IRDye680RD	926-68073	LI-COR	WB
Donkey anti-mouse IRDye680RD	926-68070	LI-COR	WB
Donkey anti-goat IRDye800CW	926-32214	LI-COR	WB
Donkey anti-mouse Alexa Fluor 647	A31571	Invitrogen	IMF
Goat anti-rabbit Alexa Fluor 647	A21244	Invitrogen	IMF
Goat anti-rabbit, Alexa Fluor 488	A11008	Invitrogen	IMF
Goat anti-mouse Alexa Fluor 488	A11001	Invitrogen	IMF
Donkey anti-goat Alexa Fluor 647	A21447	Invitrogen	IMF
donkey anti-mouse Alexa Fluor 488	A21202	Invitrogen	IMF
goat anti-rabbit IgG (Atto 594) - preabsorbed	ABIN964989	Antibodies Online	STED
goat anti-mouse IgG (Atto 647N) - preabsorbed	ABIN964964	Antibodies Online	STED

WB = Western Blot; COIP = capture antibody for co-immunoprecipitation; FC = Flow cytometry; IMF = Immunofluorescent imaging; STED = Stimulated Emission Depletion imaging

Table S2: siRNA target and oligos

**Target Gene (Protein)**,<br>Gene IDs,<br>Qiagen catalogue numbers	Target sequence (T) and<br>siRNA oligos (S = sense, A = antisense)
***TRPV2* (TRPV2),**<br>Gene accession NM_016113/gene ID: 51393,<br>SI02781359	T: 5’-CAGAGGATCTTTCCAACCACA-3’
	S: 5’-GAGGAUCUUUCCAACCACATT-3’
	A: 5’- UGUGGUUGGAAAGAUCCUCTG-3’
***TRPV1* (TRPV1),**<br>Gene accession NM_018727/gene ID: 7442,<br>SI02643480	T: 5’-CAAGTGGGACAGATTCGTCAA-3’
	S: 5’-AGUGGGACAGAUUCGUCAATT-3’
	A: 5’-UUGACGAAUCUGUCCCACUTG-3’
***CN2R* (CB$_2$R)**,<br>Gene accession NM_001841/gene ID: 1269,<br>SI05025090	T: 5’-TTGGGAGAAATCTGAGAAAGAA-3’
	S: 5’-GGGAGAAAUCUGAGAAAGAATT-3’
	A: 5’-UUCUUCUCAGAUUUCUCCCAA-3’
***CN1R* (CB$_1$R)**,<br>Gene accession NM_033181/ gene ID: 1268,<br>SI00113183	T: 5’-CAAGTTATAGTACTAGAGATA-3’
	S: 5’-AGUUAUAGUACUAGAGAUATT-3’
	A: 5’-UAUCUCUAGUACUAUAACUTG-3’
**AllStars Negative Control siRNA** (“AS” in figures),<br>Product Name: Unspecific_AllStars_1,<br>Qiagen GeneGlobe ID/catalogue number: SI03650318	
